# Synergistic Additive and Bulky Cation Engineering via a Sequential Vacuum Dry‐Dipping Process for Tin Perovskite Solar Cells

**DOI:** 10.1002/advs.76969

**Published:** 2026-10-06

**Authors:** Chun‐Hsiao Kuan, Xianyuan Jiang, Yun‐Sheng Shih, Chien‐Ling Ting, Po‐Kai Kang, Yue Lu, Sung‐Fu Hung, Hsin‐Lung Chen, Shiang Lan, Guan‐Ruei Chen, Jhih‐Min Lin, Chun‐Jen Su, Wei‐Ru Wu, Eric Wei‐Guang Diau

**Affiliations:** ^1^ Department of Applied Chemistry and Institute of Molecular Science National Yang Ming Chiao Tung University Hsinchu Taiwan; ^2^ Center For Emergent Functional Matter Science National Yang Ming Chiao Tung University Hsinchu Taiwan; ^3^ School of Physical Science and Technology ShanghaiTech University Shanghai China; ^4^ Department of Chemistry National University of Singapore Singapore Singapore; ^5^ Department of Chemical Engineering National Tsing Hua University Hsinchu Taiwan; ^6^ National Synchrotron Radiation Research Center Hsinchu Science Park Hsinchu Taiwan

**Keywords:** crystallization, energy conversion efficiency, passivation, perovskite, photovoltaics

## Abstract

Scalable fabrication of tin‐based perovskite solar cells (TPSCs) remains challenging due to rapid crystallization, poor film uniformity, and instability of Sn^2+^. Herein, we report a spin‐coating‐free approach, combining blade coating, vacuum‐drying, and controlled dipping methods, to achieve highly crystalline and uniform tin perovskite films. Through systematic studies, we reveal the universal role of SnI_2_ in governing crystallization kinetics, where pre‐formed crystalline SnI_2_ hinders cation diffusion, while amorphous SnI_2_ promotes homogeneous film growth. Additive engineering with guanidinium iodide (GAI), SnF_2_, and trimethylene sulfoxide (TMSO) improved crystallinity, suppressed Sn^2^^+^ oxidation, and extended carrier lifetimes. Moreover, we introduce n‐butylammonium tosylate (BATo) as a dual‐functional bulky cation, whose BA^+^ group enhances lattice ordering while the tosylate (TsO^−^) group passivates buried interfaces. Compared with conventional blade‐coating and spin‐coating methods, the dipping process facilitates the penetration of large molecules into the film and further enables lattice reconstruction. Devices incorporating BATo achieved a power conversion efficiency (PCE) of 10.2%, retained >90% efficiency under continuous operation, and maintained >80% after 2500 h of shelf storage. This work clarifies the intrinsic role of SnI_2_ and provides a scalable pathway toward efficient, stable, and lead‐free perovskite photovoltaics.

## Graphical abstract

A spin‐coating‐free sequential blade–dipping process is developed for efficient tin perovskite solar cells. Amorphous SnI_2_ templates are blade‐coated and vacuum‐dried, followed by a vertical dipping conversion. Enhanced by a synergistic additive matrix and bifunctional BATo cation, the resulting devices achieve a benchmark power conversion efficiency of 10.2% with exceptional long‐term stability.

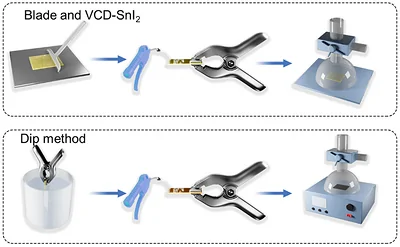

## 1 Introduction

The spin‐coating method has been widely applied to fabricate perovskite solar cells (PSCs) for the devices to achieve impressive power conversion efficiencies (PCEs) over 27% [1], highlighting its great promise in laboratory‐scale device fabrication. However, the spin‐coating method is limited to fabricating PSC devices with small areas, impeding the industrial development of large‐scale PSCs using this method. To overcome this problem, the blade‐coating method has emerged as a scalable alternative [2]. In this method, perovskite precursors dissolved in polar solvents are deposited on the substrate and then evenly spread via a doctor blade; the following solvent removal and crystallization can be performed via thermal annealing or continuous airflow. This approach has successfully enlarged the device area while maintaining high device performance, achieving PCEs up to 20% [3]. However, both blade‐ and spin‐coating processes often rely on antisolvent dripping to induce rapid nucleation. The inherent non‐uniformity of the antisolvent method and its rapid crystallization frequently lead to defective films that degrade device performance.

To address this issue, Li et al. reported in 2016 the vacuum flash‐assisted solution process (VASP) [4], which replaces the antisolvent dripping step with rapid vacuum exposure. This method effectively promotes uniform crystallization while avoiding contamination and operational complexities associated with the antisolvent method. More recently, in 2025, Yan et al. integrated blade coating with the flash process to fabricate large‐area lead‐based PSCs with enhanced uniformity, marking a major step toward scalable fabrication [5]. In addition, the dipping method has also been employed to improve film homogeneity, whereby the pre‐deposited lead precursor film is immersed in a cationic solution, allowing controlled ion exchange and uniform reaction across the substrate [6].

Despite the remarkable progress of these deposition strategies, the toxicity of lead remains a critical barrier to widespread commercialization. Consequently, tin‐based perovskites have been extensively explored as environmentally benign alternatives. The record efficiency of tin‐based PSC has reached 17% [7], demonstrating their potential for further development. Yet, intrinsic challenges such as the facile oxidation of Sn^2^^+^ to Sn^4^^+^, rapid crystallization, and defect generation severely hinder film quality and device stability. To mitigate these issues, researchers have developed many strategies, including additive engineering [8], surface passivation [9], co‐cation incorporation [10], and 2D/3D structural engineering [11]. A milestone achievement was the introduction of ethylenediammonium diiodide (EDAI_2_) as a key additive [12], which significantly boosted the device efficiencies. The incorporation of bulky organic cations such as n‐butylammonium iodide (BAI) or phenylethylammonium iodide (PEAI) leads to quasi‐2D Ruddlesden–Popper (RP) perovskite structures that suppress Sn^2^^+^ oxidation [13], while isopropylammonium iodide (IMI) and GAI form co‐cation perovskites that tune the Goldschmidt tolerance factor and stabilize the lattice [8, 14]. Furthermore, passivation molecules such as phenylhydrazinium iodide (PHSCNI) effectively reduce surface Sn^4^^+^ [9], and diammonium salts such as (aminomethyl)piperidinium (4AMP) can form the Dion–Jacobson (DJ) phase that retarded the crystallization [15].

Most of these advances, however, rely on conventional one‐step spin‐coating method, which imposes severe constraints when thicker films are desired, as the inevitable rapid antisolvent quenching accelerates crystallization and yields poor film morphology [16]. To circumvent this, we proposed a two‐step deposition route in which SnI_2_ is first deposited as a precursor layer, followed by slow diffusion of organic cations using a co‐solvent system involving isopropanol (IPA), hexafluoro‐2‐propanol (HFP), and chlorobenzene (CB) [17]. This approach effectively suppresses crystallization kinetics and improves film quality. Nonetheless, the second step still relies on spin coating, where differences in cation diffusion rates and the rapid evaporation of solvents frequently lead to film inhomogeneity. Thus, the scalable tin‐based perovskite fabrication via the spin‐coating method remains highly challenging.

Inspired by the two‐step strategy, we developed a sequential blade‐coating/flash‐drying/dipping process for fabrication of the tin‐based perovskite layer (the fabrication videos are shown in Supporting Information). Such an approach could provide the necessary loading time for cation infiltration while avoiding the concentration gradients and rapid solvent evaporation inherent to spin coating. Importantly, it is well known that annealing of the SnI_2_ precursor in the first step should be avoided; the underlying mechanism has remained unclear [17, 18, 19]. Here, we systematically investigated the crystallization kinetics using atomic force microscopy (AFM), small‐angle X‐ray scattering (SAXS), and in situ grazing‐incidence wide‐angle X‐ray scattering (GIWAXS) techniques. We found that annealed SnI_2_ quickly forms dense granular crystallites that hinder subsequent cation incorporation, while vacuum‐dried (VCD)‐SnI_2_ films remain uniform and thus promote homogeneous perovskite formation. Time‐of‐flight secondary ion mass spectrometry (TOF‐SIMS) revealed that dipping results in profoundly different ion distributions compared to spin coating. We attribute this to the fact that in spin coating, the rapid drying of the cation solution limits additive or co‐cation penetration into the perovskite bulk, often leaving them aggregated near the surface.

To further improve the device performance for TPSCs, we incorporated a novel pseudo‐halogen 2D ammonium salt, n‐butylammonium tosylate (BATo), as a bifunctional additive for surface passivation. SIMS analyses confirmed that the BA cation enhances crystallinity, while the tosylate anion (TsO^−^) effectively passivates the buried interface. Additionally, we introduced tetramethylene sulfoxide (TMSO) as an additive to further retard the crystallization rate in solution [20, 21]. Density functional theory (DFT) calculations and X‐ray absorption spectroscopy (XAS) analyses revealed that SnI_2_ readily coordinates with the nitrogen of FAI, forming disordered polycrystalline domains with uncontrollable growth kinetics. In contrast, the BATo‐blade‐coating/flash‐drying/dipping process establishes strong coordination between BATo and FAI, stabilizes SnI_2_ orientation via TMSO, and gradually leads to the formation of uniform and highly oriented tin perovskite films. Ultimately, this blade‐dip two‐step strategy enabled the TPSC device to achieve a PCE of 10.2% without using the traditional spin‐coating method. This work demonstrates a novel sequential tin‐based perovskite deposition route beyond spin coating with synergistic additives to improve the device performance. It not only advances fundamental understanding of SnI_2_ crystallization kinetics but also provides a reliable blueprint for scalable, lead‐free perovskite solar cell manufacturing.

## 2 Results and Discussion

### 2.1 Structural Evolution of SnI_2_ Precursor Films

To elucidate the role of SnI_2_ in dictating crystallization kinetics during tin‐based perovskite formation, we first investigated the structural evolution of SnI_2_ precursor films under different processing conditions. SnI_2_ is an indispensable component in both tin‐only and Sn–Pb perovskites, yet it is also responsible for its characteristically rapid crystallization, which severely limits film quality and scalability. We therefore compared two types of SnI_2_ precursor films: one prepared by vacuum drying without additional thermal treatment (hereafter referred to as “VCD”), and the other subjected to mild thermal annealing at 70°C (referred to as “annealed”). Their structural and morphological characteristics were systematically analyzed by GIWAXS, grazing‐incidence small‐angle X‐ray scattering (GISAXS), and AFM techniques.

GIWAXS analysis revealed striking differences in crystallinity between the two films (Figure 1a,b). The VCD‐SnI_2_ film displayed broad and diffuse scattering halos, indicative of an amorphous or weakly ordered structure. In contrast, the annealed film exhibited sharp Bragg reflections and intense diffraction rings, characteristic of highly crystalline SnI_2_ domains. The corresponding 1D profiles (Figure S1) further confirmed the emergence of long‐range order upon annealing. These results indicate that thermal treatment triggers a substantial rearrangement of SnI_2_ molecules into dense crystallites. The GISAXS profiles corroborated the crystallization behavior (Figure 1c). The VCD‐film showed no discernible scattering peaks, reflecting its disordered microstructure. In contrast, the annealed film exhibited a pronounced correlation peak at q ∼0.078 Å^−^^1^, corresponding to an average interparticle spacing of ∼7.9 nm. This feature unambiguously signifies the formation of nanocrystalline SnI_2_ domains with a regular spatial arrangement. In situ GIWAXS tracked the crystallization kinetics of the SnI_2_ film during thermal annealing (Figure 1d). An initial induction period shows weak, diffuse scattering with a broad low‐q feature (q ≈ 0.35–0.36 Å^−^^1^), consistent with an amorphous ordered state. A rapid nucleation event then occurs at 90–100 s, evident from a transient vertical intensity spike in the q–t map. Immediately thereafter, a narrow, intense horizontal band emerges at q = 0.92–0.94 Å^−^^1^ and persists through the remainder of the anneal with steadily increasing intensity while its position remains essentially fixed. This behavior indicates fast formation of a dominant crystalline phase followed by grain ripening with minimal lattice parameter change. Concomitantly, the low‐q feature decays, implying consumption of the amorphous/intermediate species. No additional long‐lived peaks are detected, suggesting that the annealing predominantly yields a single, highly crystalline SnI_2_ phase.

**FIGURE 1:**
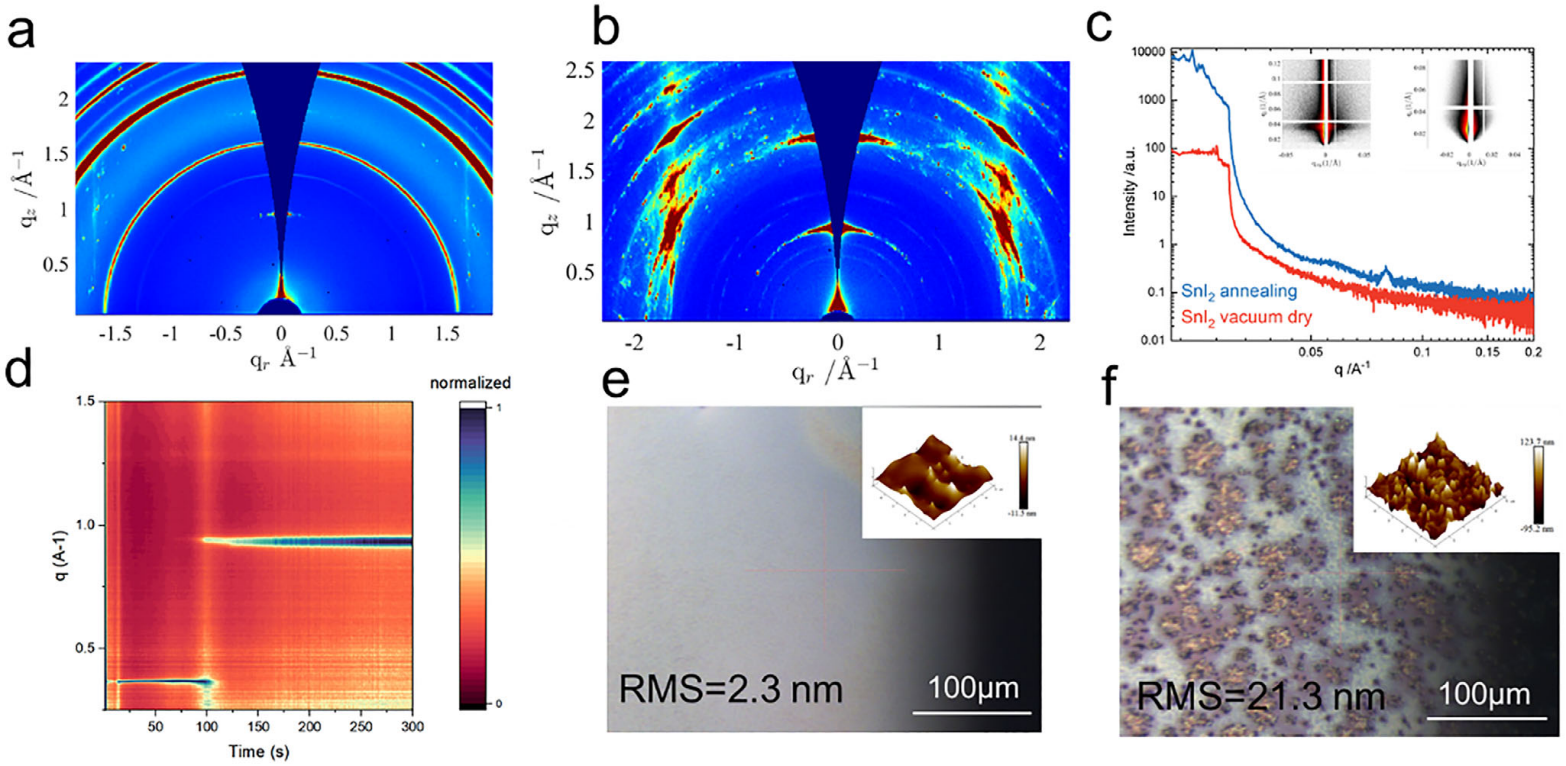
GIWAXS 2D patterns of (a) VCD and (b) annealed SnI_2_ films. (c) GISAXS intensity profiles of vacuum‐dried and annealed SnI_2_ films as indicated. The corresponding 2D scattering patterns are shown in the insets. The annealed film exhibits a distinct scattering peak, attributed to the formation of SnI_2_ crystallites. The regular spatial arrangement of these crystallites gives rise to a characteristic correlation feature in the GISAXS profiles. (d) In situ GIWAXS of VCD‐SnI_2_ during thermal annealing at 70°C. Tapping‐mode AFM images of (e) VCD and (f) annealed SnI_2_ films. The VCD‐film exhibits a smooth surface with no discernible phase‐separated features and an RMS roughness of approximately 2.3 nm. In contrast, the annealed film shows a significantly rougher and more heterogeneous surface, with an RMS roughness of 21.3 nm.

Complementary AFM imaging provided real‐space evidence of the morphological transitions (Figure 1e,f). The VCD‐SnI_2_ surface was uniform and smooth, with a root‐mean‐square (RMS) roughness of only 2.3 nm, consistent with its amorphous nature. By contrast, the annealed film exhibited pronounced surface heterogeneity and granular features, with an RMS roughness of 21.3 nm. These morphological changes are fully consistent with the formation of densely packed SnI_2_ crystallites during annealing.

Collectively, these results demonstrate that VCD‐SnI_2_ films retain an amorphous or weakly ordered character that is highly reactive and thus more favorable for subsequent perovskite conversion. Such a structure facilitates homogeneous cation interdiffusion and uniform nucleation, yielding continuous and defect‐minimized perovskite films. In contrast, annealed SnI_2_ films are dominated by pre‐formed crystalline domains, which not only restrict ionic diffusion but also act as physical barriers that disrupt grain continuity during perovskite growth. These domains may further induce phase segregation and incomplete conversion, leading to non‐uniform perovskite films and degraded device performance.

### 2.2 Blade–Dipping Process and Molecular Engineering of Bulky Organic Cations

To integrate the advantages of the two‐step deposition method while addressing the challenges of rapid SnI_2_ crystallization, we developed a blade–dipping process that combines procedures of blade coating, vacuum drying, and liquid‐phase dipping (Figure 2a). In the first step, SnI_2_ precursor films were deposited via blade coating and immediately subjected to nitrogen gas flow to remove residual solvent, followed by rapid vacuum flash‐drying without thermal annealing. This treatment yields a uniform SnI_2_ intermediate layer that avoids the formation of large crystalline domains, thereby providing a reactive yet homogeneous template for subsequent perovskite conversion. In the second step, the pre‐dried SnI_2_ films were immersed in a mixed cation solution containing formamidinium iodide (FAI) and selected bulky organic cations, dissolved in a 1:1 mixture of IPA and HFP. The reduced polarity of this solvent mixture prevents rapid dissolution of the perovskite lattice, while simultaneously offering a homogeneous medium for ion diffusion and reaction, thus ensuring uniform perovskite crystallization. After dipping, the films were subjected to nitrogen drying and a mild post‐annealing step.

**FIGURE 2:**
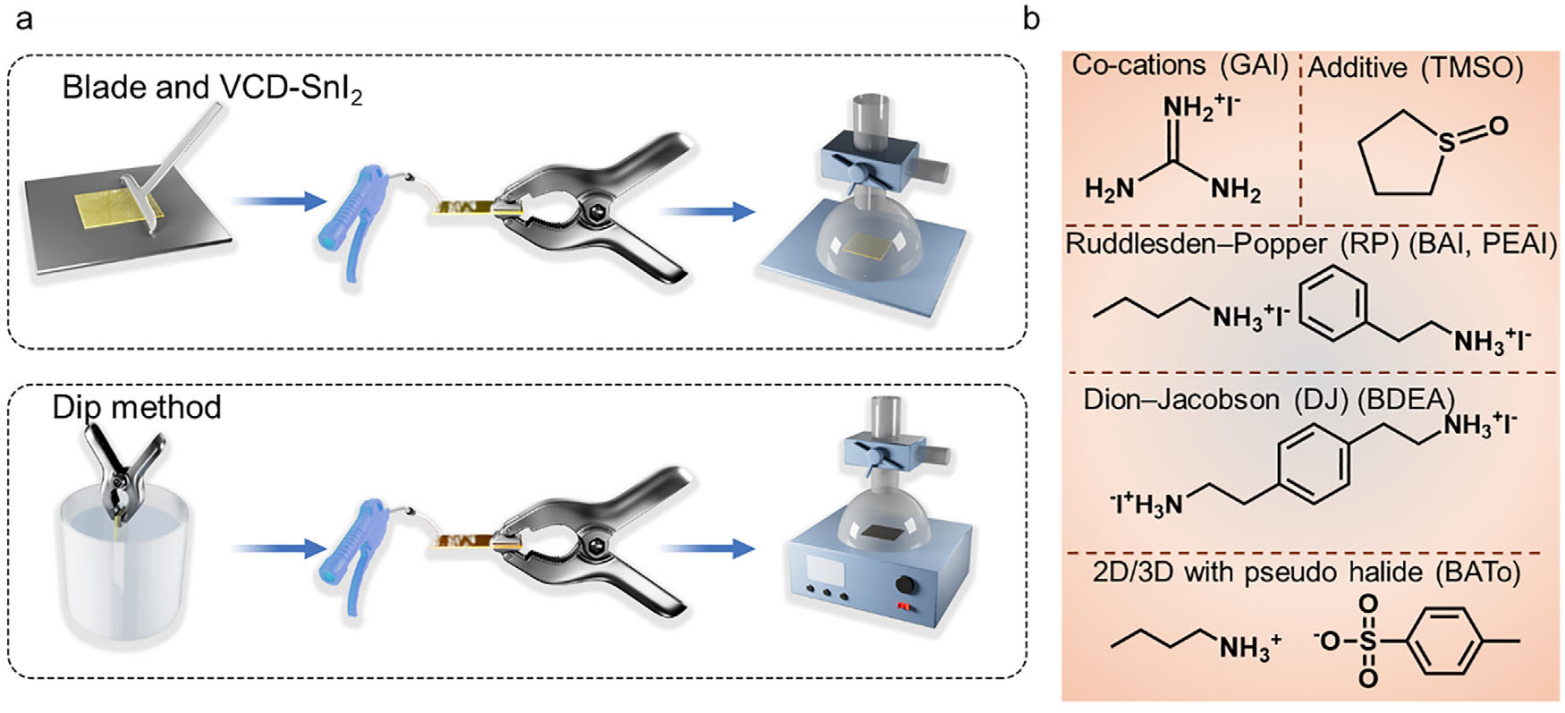
(a) Schematic of the blade–dipping process. (b) Co‐cations and additives which added in the A/FA solution in the second step or SnI_2_ in the first step.

We systematically optimized the reaction parameters, including the concentration of SnI_2_ precursors and the loading time during dipping (details are provided in the Figures S2–S49). In particular, GAI and SnF_2_—a well‐established co‐cation and an additive in tin‐based perovskites—were revisited and finely tuned under the blade–dipping framework, yielding improved crystallization control and film uniformity. While 3D tin perovskites (e.g., FASnI_3_) exhibit promising optoelectronic properties, they are intrinsically unstable in ambient conditions due to facile Sn^2^^+^ oxidation and high moisture sensitivity. Introducing bulky organic cations enables the formation of 2D (e.g., Ruddlesden–Popper, RP) or quasi‐2D/3D structures, which mitigate oxidation and enhance stability. However, excessive 2D incorporation can generate insulating layers that hinder charge transport. Therefore, a rational balance between dimensionality and film uniformity is required. Combining scalable blade–dip processing with controlled incorporation of large organic cations provides a pathway to achieve high stability, reproducibility, and efficiency in large‐area tin perovskite devices. In this context, we investigated the role of four representative bulky cations with distinct structural motifs (Figure 2b): PEAI–a widely used RP‐type cation with a phenyl group; BAI–a linear alkyl ammonium cation commonly applied in RP perovskites; 1,4‐benzenediethanamine dihydroiodide (BDEA)–a diammonium species with an aromatic core, corresponding to a DJ‐type spacer; BATo–a newly designed pseudo‐halide cation combining the butylammonium backbone of BA^+^ cation with the TsO^−^ anion, which has never been reported for TPSC. The sulfonate group (─SO_3_^−^) offers strong coordination with Sn^2^^+^ (via S═O → Sn^2^^+^ interaction), enabling both defect passivation at iodide vacancies and enhanced lattice stabilization.

To further enhance perovskite crystallization, TMSO was introduced in the first step as an additive to the SnI_2_ precursor [21]. Inspired by its role in lead‐based perovskites, where it promotes stable intermediates, large‐grain growth, and defect reduction, TMSO is expected to coordinate with under‐coordinated Sn^2^^+^, suppress oxidation, and enable controlled crystal nucleation and growth. To the best of our knowledge, this is the first example of using a TMSO additive in tin‐based perovskite systems.

By fixing the bulky cation content at 10 mol% (replacing PEAI) in the dipping solution and optimizing the TMSO amount at 15 µL in the first step, we achieved reproducible and relatively stable device performance. Comparative studies of BAI, BDEA, and BATo demonstrated distinct influences on film crystallization and optoelectronic quality in the second step. Notably, BATo exhibited superior modification effects, attributed to its dual functional nature, which provides both structural templating from the BA^+^ cation and chemical passivation from the TsO^−^ pseudo‐halide group. This dual‐mode stabilization strategy effectively improves lattice order, suppresses Sn^2^^+^ oxidation, and ensures interfacial integrity, ultimately yielding tin perovskite films with enhanced crystallinity, reduced defect density, and superior photovoltaic performance.

### 2.3 Film Characterizations

To investigate the incorporation behavior of guanidinium (GA^+^) in tin perovskites, we first analyzed the depth distribution using TOF‐SIMS. In the conventional two‐step process where GA^+^ is introduced in the second‐step solution, the SIMS profiles reveal that GA^+^ predominantly accumulates at the top and bottom regions of the perovskite film [22]. This indicates that GA^+^ primarily functions to passivate surface and buried interface defects. In contrast, as shown in Figure 1a, in the blade–dipping process, GA^+^ shows a markedly different distribution: GA^+^ is uniformly dispersed throughout the perovskite layer within a depth of ∼20 nm, with significant signals even at the buried interface. We attribute this to the liquid‐phase environment, where both GA^+^ and FA^+^ cations are present in a polar medium that promotes homogeneous crystallization. The enrichment of GA^+^ at the bottom of the film stabilizes the buried perovskite/HTL interface and enhances defect passivation.

We further compared the spatial distribution of four representative bulky cations—PEAI, BAI, BATo, and BDEA—using TOF‐SIMS (Figure 3b–e). Interestingly, despite their bulky aromatic structures, both PEA^+^ and BDEA were detected throughout the perovskite film, not only at the surface, suggesting partial incorporation into the A‐site lattice. However, their behaviors differ: BDEA exhibited weak and irregular signals across the film depth (Figure 3d), indicating poor incorporation and limited passivation capability. In contrast, both PEA^+^ (Figure 3b) and BA^+^ (Figure 3c) ions displayed depth‐dependent profiles, with moderate incorporation in the mid‐layer and strong accumulation at the bottom interface, implying an effective buried interfacial passivation effect. For BATo (Figure 3e), a unique distribution was observed: while the BA^+^ cations participated in A‐site incorporation, the TsO^−^ anions were mainly detected at the top and bottom interfaces, but absent in the mid‐layer (Figure 3e). This suggests that BATo operates in a dual‐functional manner—BA^+^ contributes to lattice incorporation and defect suppression, while TsO^−^ simultaneously passivates both the film surface and the buried interface. The 3D SIMS depth profiles (Figure 3f–j) further confirm these observations, highlighting the distinct incorporation and passivation mechanisms of each bulky organic cation. To further verify the buried‐interface enrichment of TsO^−^, FTIR and EDS analyses were performed. The S═O vibration of BATo exhibits a redshift from 1181.6 to 1179.5 cm^−^^1^ on PEDOT:PSS (Figure S59a), indicating specific interfacial interactions. Meanwhile, sulfur EDS mapping of the exposed buried interface (Figure S59b) reveals pronounced S accumulation at the bottom surface, consistent with the TOF‐SIMS results (Figure 3e). These observations confirm that TsO^−^ diffuses through the amorphous SnI_2_ template and preferentially anchors at the PEDOT:PSS/perovskite interface.

**FIGURE 3:**
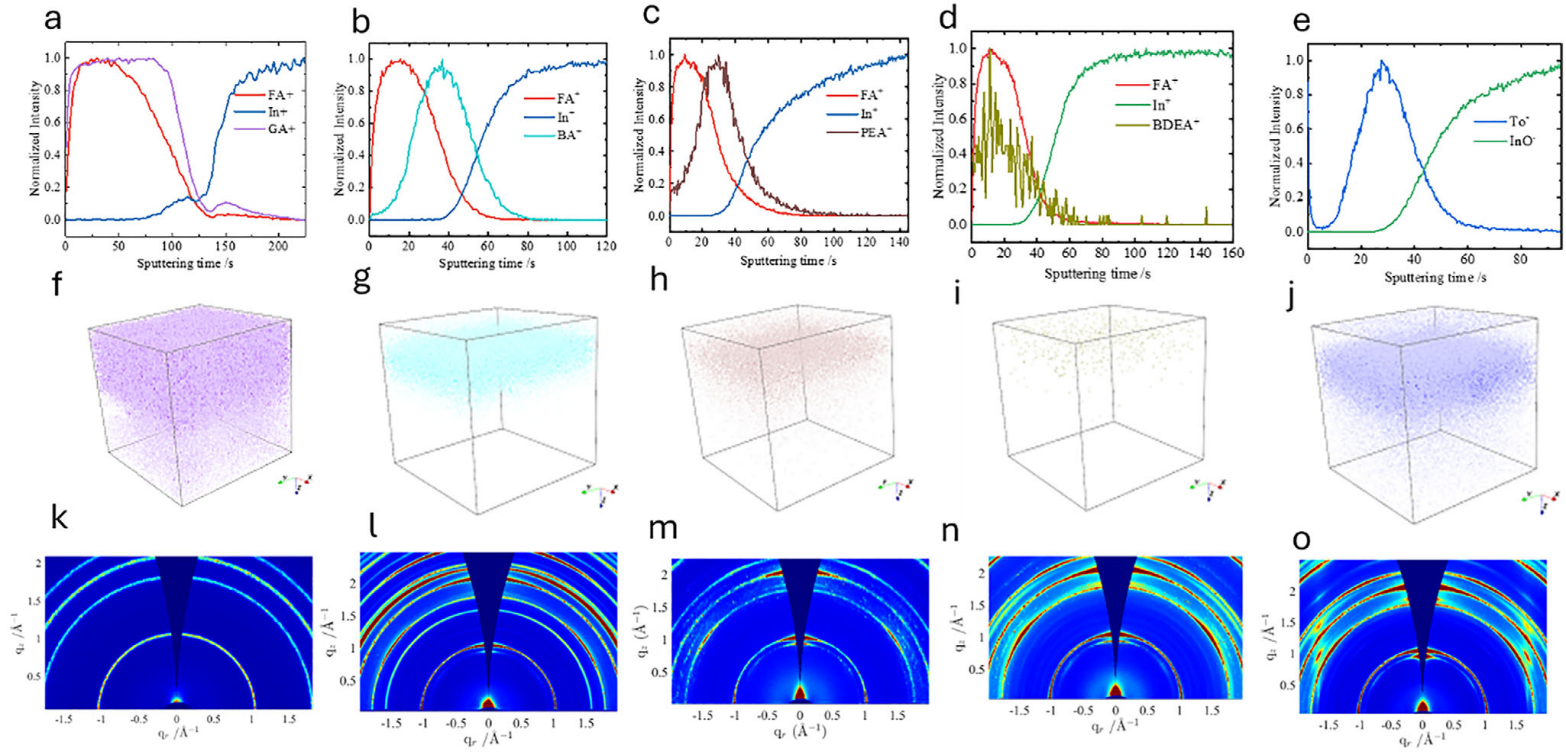
TOF‐SIMS, 3D profiles and GIWAXS images of tin perovskites fabricated with (a,f,k) FA/GA, (b,g,l) FA/BA, (c,h,m) FA/PEA, (d,i,n) FA/BDEA and (e,j,o) FA/BATo made by using the blade–dipping process.

The structural crystallizations of the perovskites fabricated using these organic cations were examined by the GIWAXS images (Figure 3k–o). For PEAI‐modified films, scattering was mainly concentrated along the q_z_ axis, showing weak preferential orientation but with diffuse and low‐intensity rings, indicating small grain sizes or poor crystallinity. BDEA‐modified films exhibited more uniform scattering with strong vertical and diagonal features, suggesting good crystallinity with partial orientation. BATo‐modified films showed the most pronounced scattering pattern, with bright striped rings and distinct diffraction spots along multiple directions, signifying strong crystallinity, high ordering, and mixed orientations. In contrast, BAI‐modified films displayed concentric circular rings with evenly distributed intensity, indicative of high crystallinity but with largely random grain orientation. Notably, in the BATo‐based perovskite, we observed strong GIWAXS features consistent with highly oriented SnI_2_ domains, which help with the following crystallization. Previous reports have shown that degraded tin perovskites typically exhibit poorly oriented SnI_2_ upon phase separation [23]. In our blade–dipping process, however, the presence of BATo not only slowed down the crystallization kinetics but also enabled SnI_2_ to act as an oriented template, guiding the growth of highly oriented tin perovskite phases. This synergistic effect underlies the superior crystallinity and stability of the BATo‐modified films.

To clarify whether BATo induces a 2D RP phase, low‐angle XRD measurements were performed. As shown in Figure S60, no diffraction peaks characteristic of n = 1 or n = 2 RP phases are observed, while only the 3D FASnI_3_ (100) reflection appears at ≈14°. This observation is consistent with the GIWAXS results (Figure 3o), which show no low‐q RP stacking features, confirming the absence of a long‐range ordered 2D phase.

Our experimental findings demonstrated that the incorporation of BATo molecules significantly improves the crystallinity and enhances the preferential orientation of tin‐based perovskite films. To rationalize these effects, we conducted DFT calculations of charge density distributions and binding energies (Figure 4a,b). The results show that the binding energy of BATo–FA is considerably greater than that of FA–SnI_2_, while the interaction between BATo and SnI_2_ is also stronger compared to FA–SnI_2_. These results suggest that during crystallization, BATo preferentially interacts with both FA and SnI_2_ to form a stable BATo–FA–SnI_2_ intermediate complex. These intermediates undergo retarded formation of FASnI_3_, thereby slowing down crystallization kinetics and promoting oriented crystal growth, consistent with the GIWAXS patterns shown in Figure 3o.

**FIGURE 4:**
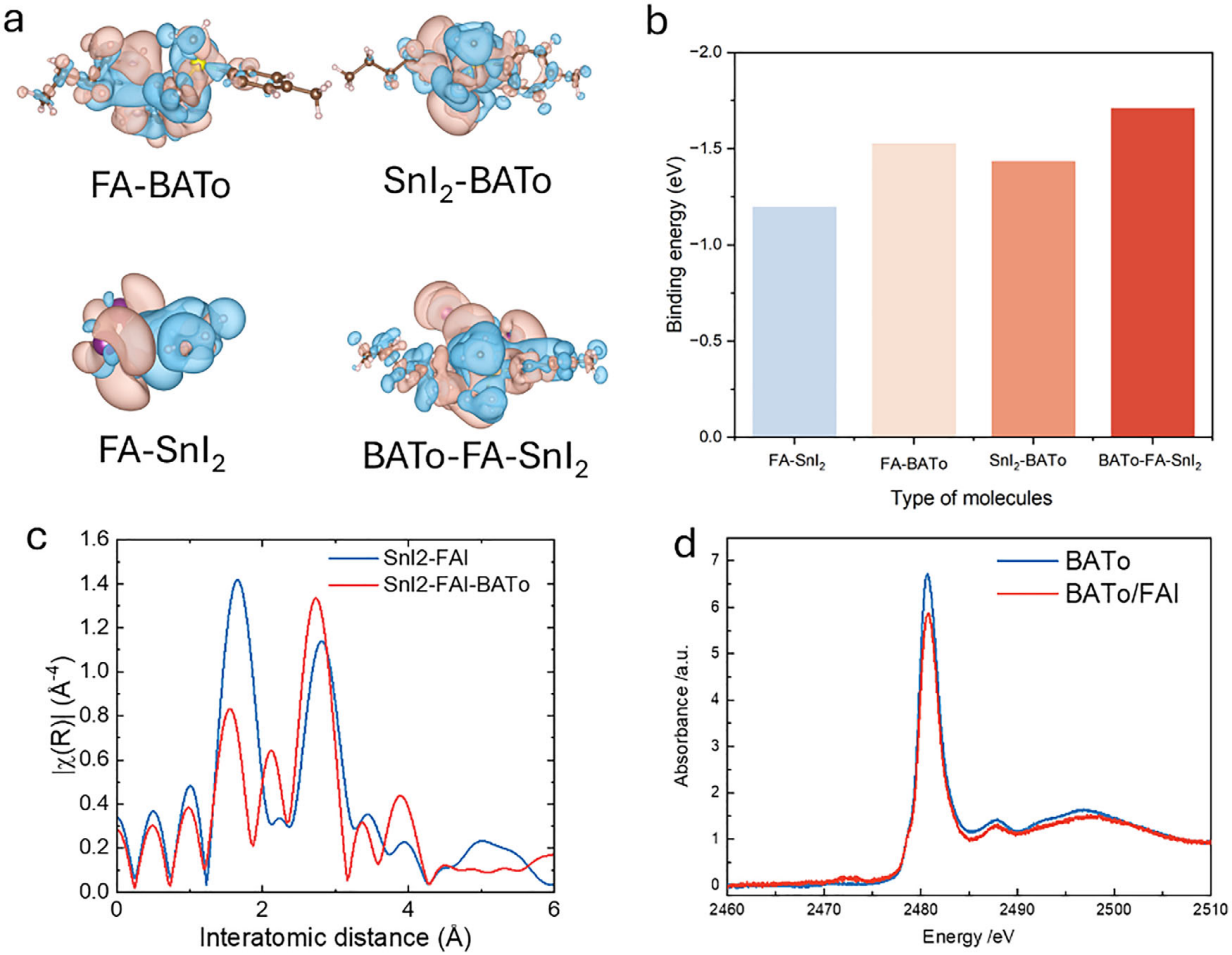
(a) Distributions of the charge density obtained from DFT calculations and (b) binding energies of FA‐SnI_2_, FA‐BATo, SnI_2_‐BATo, and BATo‐FA‐SnI_2_. (c) S tender X‐ray absorption of BATo and BATo/FAI, (d) Sn hard X‐ray absorption of SnI_2_‐FAI and SnI_2_‐FAI‐BATo.

DFT calculations further reveal that TMSO exhibits a stronger coordination interaction with Sn^2^^+^ than DMSO and DMF (Figure S61 and Table S2), indicating a greater capability to stabilize Sn^2^^+^ and suppress oxidation during crystallization.

To gain further mechanistic insight, we performed XAS at the Sn K‐edge and S K‐edge to probe the local coordination environment. The Sn K‐edge spectra (Figure 4c) confirmed a pronounced Sn─N coordination pathway between SnI_2_ and FAI, reflecting the strong interaction responsible for rapid growth of the tin perovskite films. However, upon incorporation of BATo, the Sn─N coordination intensity decreased significantly, indicating weakened interaction between SnI_2_ and FAI. This suggests that the presence of BATo disrupts the direct Sn─N coordination, thereby retarding crystallization for the formation of uniform perovskite films. Complementary evidence was obtained from S K‐edge XAS measurements (Figure 4d). A notable reduction in the white‐line intensity was observed in the BATo–FAI complex compared to pristine BATo, implying electron transfer into the sulfur valence orbitals of the sulfonyl group. This result indicates that the sulfonyl moiety of BATo preferentially coordinates with the amino group of FAI, rather than engaging in intramolecular interactions. Such preferential coordination effectively competes with the Sn─N interaction, thereby suppressing the direct SnI_2_–FAI reaction to retard the crystal growth. Considered all together, the combined DFT and XAS analyses provide strong mechanistic evidence that BATo molecules introduce a competing coordination pathway via the sulfonyl group, leading to the formation of a BATo–FA–SnI_2_ intermediate. This intermediate state reduces Sn─N coordination, moderates crystallization kinetics, and guides the oriented growth of tin perovskites, ultimately accounting for the superior structural quality observed experimentally.

We carried out further investigations for the tin perovskite produced using four different bulky organic cations (PEAI, BAI, BDEA and BATo). Morphological analyses confirmed that all cations produced dense, pinhole‐free films (Figure S62), indicating effective defect passivation in the presence of TMSO. AFM results revealed smooth surfaces with roughness below 20 nm, with the BATo sample yielding the lowest roughness (RMS 19.2 nm) (Figure S63). Interestingly, BATo films appeared thicker than those with PEAI, potentially influencing absorbance and current density. XRD patterns (Figure 5a and Figure S64) showed no change in crystal orientation but exhibited peak shifts associated with cation size. BDEA, the largest cation, caused a shift to lower angles (lattice expansion), whereas smaller BA^+^ induced contraction of the crystal size. BATo, whose tosylate anion did not enter the lattice, displayed the strongest peak intensity, signifying improved crystallinity. This trend was corroborated by the TEM image of the BATo sample (Figure 5b), which confirmed well‐defined lattice fringes with d‐spacings of 2.71–2.75 Å, consistent with the cubic perovskite planes.

**FIGURE 5:**
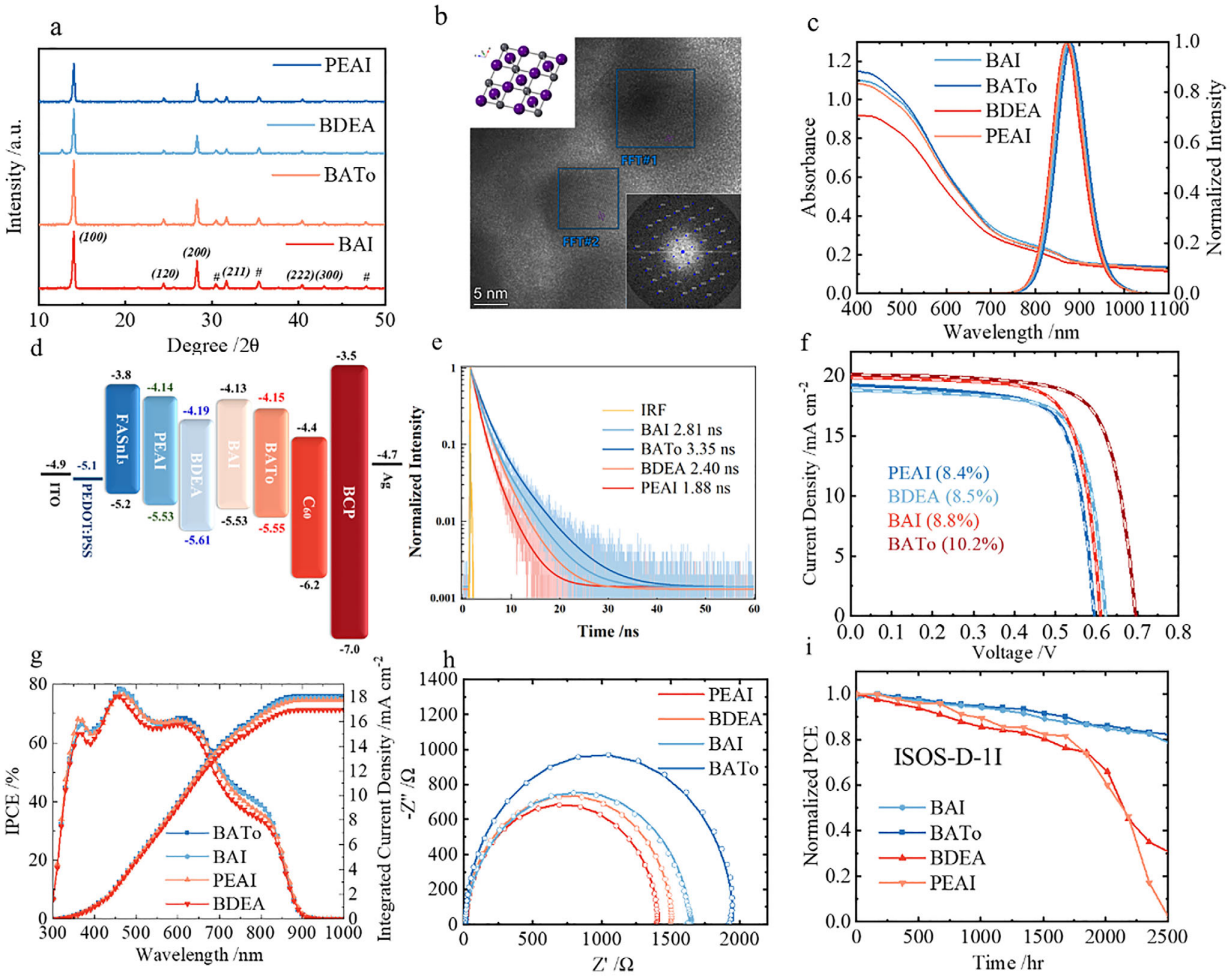
(a) XRD patterns of tin perovskite films incorporating different bulky organic cations. (b) TEM image of a representative BATo sample, with corresponding FFT patterns. (c) UV–vis absorption and photoluminescence (PL) spectra of the perovskite films prepared with different bulky organic cations. (d) Energy‐level diagram of perovskite films with different cation compositions, derived from UPS and optical bandgap measurements. (e) TCSPC spectra under different large organic cation conditions. (f) Current density–voltage (*J–V*) curves of devices fabricated with different bulky cations, showing negligible effect of hysteresis. (g) Incident photon‐to‐current efficiency (IPCE) spectra of the corresponding devices. (h) Electrochemical impedance spectroscopy (EIS) Nyquist plots, highlighting recombination resistance under different conditions. (i) Long‐term stability statistics of devices incorporating different bulky organic cations, measured under inert glovebox conditions.

Optical measurements revealed that BATo‐modified films have the highest absorption, followed by BAI and PEAI, while BDEA films showed reduced absorption (Figure 5c). Tauc plots confirmed similar bandgaps (1.39–1.40 eV) for most samples, except for BDEA, which exhibited a slightly blueshift (∼1.42 eV) due to lattice distortion (Figure S65). PL spectra were centered around 880 nm across all samples, verifying consistent bandgaps, but the BATo film exhibited the strongest intensity with a slight redshift, indicating defect suppression and improved non‐radiative recombination. Electronic characterization further highlighted the beneficial role of BATo. UPS results of BATo (Figure 5d and Figure S66) showed a reduced work function and an upward Fermi‐level shift, suggesting weaker p‐type character and enhanced electron conductivity. KPFM measurements (Figure S67) reveal a more homogeneous surface potential distribution for the BATo‐modified film, indicating reduced defect‐induced potential fluctuations and suppressed charge trapping. These shifts provided more favorable band alignment with charge transport layers, facilitating efficient charge extraction for the BATo sample. Carrier dynamics (PL lifetimes) measured by TCSPC (Figure 5e) followed the trend BATo >BAI >BDEA >PEAI, consistent with reduced surface/interfacial defects for retarded charge recombination. Consistent with the reduced potential fluctuations observed by KPFM, TCSPC and device recombination analyses (Figure S68) reveal the longest carrier and recombination lifetimes for the BATo‐modified sample, indicating effective suppression of non‐radiative recombination. XPS analysis confirmed effective suppression of Sn^2+^ oxidation by all bulky cations, with BATo achieving the lowest Sn^4+^ content (Figure S69 and Table S11).

### 2.4 Device Characterizations

Device‐level studies clearly demonstrated the superior performance of BATo (Figure 5f,g). The detailed photovoltaic parameters of the perovskite solar cells treated with different bulky cations, including average and champion values, are systematically summarized in Table 1. Statistical analysis of 15 devices per condition (Figure S70) revealed that BATo achieved the highest efficiency (10.2%), outperforming PEAI (8.4%), BDEA (8.6%), and BAI (8.8%). Enhanced performance of the BATo device originated from simultaneous improvements in *J*_SC_ (∼20 mA cm^−^^2^), *V*_OC_ (∼0.70 V), and FF (>70%). All devices exhibited negligible hysteresis, confirming stable operation. EQE (IPCE) spectra corroborated the photocurrent trends, with the integrated values matching the *J–V* results. Charge transport and stability further reinforced the advantage of BATo. EIS measurements (Figure 5h) revealed the largest recombination resistance, consistent with prolonged carrier lifetimes obtained from TCSPC. Long‐term stability testing under inert conditions (Figure 5i and Figure S71) showed that BATo‐ and BAI‐based devices retained over 80% of their initial efficiency after 2500 h, while PEAI degraded almost completely and BDEA retained only ∼30%. These results highlight that BATo not only improves device performance but also substantially enhances operational durability. In summary, replacing PEAI with alternative bulky cations revealed significant differences in perovskite crystallinity, carrier dynamics, device performance, and stability. Among these candidates, BATo provided the most favorable combination of morphology, optical absorption, band alignment, defect passivation, and stability. This synergy enabled both high efficiency (10.2%) and long‐term durability, establishing BATo as a promising design principle for advancing stable tin‐based perovskite solar cells fabricated using the newly developed sequential blade‐dip coating technique. The scalability of the blade–dipping process was further demonstrated using a 5 × 5 cm^2^ perovskite film (Figure S72). PCE mapping of a 4 × 4 sub‐cell array revealed excellent uniformity, with ∼88% of the devices exhibiting efficiencies above 9%. Moreover, a large‐area device (∼2.6 cm^2^) achieved a PCE of 8.46% (Figure S73), confirming the scalability of this deposition strategy.

**TABLE 1:** Photovoltaic parameters (V_OC_, J_SC_, FF, and PCE) of the perovskite solar cells treated with different bulky cations (PEAI, BAI, BDEA, and BATo). The data are presented as the mean ± standard deviation, calculated from 15 individual devices for each condition, alongside the champion PCE. All devices were measured with an active area of 0.1 cm^2^ under a reverse voltage scan.

Bulky cation	V_OC_ (V)	J_SC_ (mA cm^−2^)	FF (%)	PCE (%)	Champion PCE (%)
PEAI	0.596 ± 0.015	19.355 ± 0.248	70.48 ± 1.62	8.13 ± 0.13	8.4
BAI	0.607± 0.012	19.780 ± 0.148	72.14 ± 1.23	8.66 ± 0.13	8.8
BDEA	0.618± 0.014	18.936 ± 0.161	70.94 ± 1.86	8.29 ± 0.16	8.6
BATo	0.643± 0.038	19.673 ± 0.188	71.28 ± 1.22	9.02 ± 0.52	10.2

## 3 Conclusion

We developed a two‐step blade–dip strategy that yields uniform, defect‐suppressed tin perovskite films without involving spin coating. In the first step with blade coating of SnI_2_, we found the general role of SnI_2_ in tin perovskite crystallization: crystalline SnI_2_ produced by thermal annealing forms diffusion barriers, while amorphous SnI_2_ formed by flash vacuum drying enables homogeneous cation incorporation. Following this approach, additive engineering was applied in the second step with dip coating by introducing TMSO as an additive and BATo as a bifunctional bulky organic cation to produce highly crystalline, defect‐passivated, and uniform perovskite films with superior stability; other bulky organic cations like PEAI, BAI, and BDEA were also applied for comparison. The BATo device achieved a champion efficiency of 10.2% and maintained >80% of performance after 2500 h of shelf storage. This study not only demonstrates a scalable route for lead‐free perovskite photovoltaics but also establishes the mechanistic framework of modulating SnI_2_ as a universal regulator for retarded crystallization to grow a high‐performance tin perovskite film.

## 4 Experimental Methods

Details of chemicals, film preparation and characterizations, device fabrication and optimization, supplementary figures and tables are provided in Supporting Information. The video for fabricating the device using the proposed dip coating procedure is provided as an MP4 file.

## Author Contributions

**Xianyuan Jiang**: conceptualization, methodology, investigation, validation, formal analysis, Writing – original draft. **Chun‐Hsiao Kuan**: conceptualization, methodology, writing – original draft, investigation, validation, formal analysis. **Yue Lu**: software, investigation. **Chien‐Ling Ting**: data curation, investigation, formal analysis. **Po‐Kai Kang**: investigation, formal analysis, data curation. **Hsin‐Lung Chen**: investigation, validation. **Guan‐Ruei Chen**: investigation, validation. **Sung‐Fu Hung**: investigation, validation. **Yun‐Sheng Shih**: conceptualization, methodology, investigation, validation, writing – original draft, formal analysis. **Chun‐Jen Su**: investigation, validation. **Shiang Lan**: methodology, investigation. **Jhih‐Min Lin**: investigation, validation. **Eric Wei‐Guang Diau**: conceptualization, methodology, supervision, funding acquisition, visualization, project administration, resources, writing – review and editing. **Wei‐Ru Wu**: investigation, validation.

## Conflicts of Interest

The authors claim no conflicts of interest in this work.

## Supporting information




**Supporting File 1**: advs76969‐sup‐0001‐SuppMat.docx.

**Supporting File 2**: advs76969‐sup‐0002‐VideoS1.docx.

## Data Availability

The data that support the findings of this study are available on request from the corresponding author. The data are not publicly available due to privacy or ethical restrictions.

## References

[1] National Laboratory of the Rockies (NLR) , Best Research‐Cell Efficiency Chart, 2026, accessed August, 2026, https://www.nlr.gov/pv/cell‐efficiency.

[2] Y. Deng , E. Peng , Y. Shao , Z. Xiao , Q. Dong , and J. Huang , “Scalable Fabrication Of Efficient Organolead Trihalide Perovskite Solar Cells With Doctor‐Bladed Active Layers,” Energy & Environmental Science 8 (2015): 1544–1550, 10.1039/C4EE03907F.

[3] Y. Deng , X. Zheng , Y. Bai , Q. Wang , J. Zhao , and J. Huang , “Surfactant‐Controlled Ink Drying Enables High‐Speed Deposition Of Perovskite Films For Efficient Photovoltaic Modules,” Nature Energy 3 (2018): 560–566, 10.1038/s41560-018-0153-9.

[4] X. Li , D. Bi , C. Yi , et al., “A Vacuum Flash–Assisted Solution Process For High‐Efficiency Large‐Area Perovskite Solar Cells,” Science 353 (2016): 58–62, 10.1126/science.aaf8060. 27284168

[5] B. Yan , W. Dai , Z. Wang , et al., “3D Laminar Flow–Assisted Crystallization Of Perovskites For Square Meter–Sized Solar Modules,” Science 388 (2025): adt5001, 10.1126/science.adt5001. 40403065

[6] M. Adnan and J. K. Lee , “All Sequential Dip‐Coating Processed Perovskite Layers From an Aqueous Lead Precursor for High Efficiency Perovskite Solar Cells,” Scientific Reports 8 (2018): 2168, 10.1038/s41598-018-20296-2. PMC5794860 29391423

[7] T. Li , X. Luo , P. Wang , et al., “Tin‐Based Perovskite Solar Cells With A Homogeneous Buried Interface,” Nature 648 (2025): 84–90. 10.1038/s41586-025-09724-2 41092964

[8] C.‐H. Kuan , J.‐M. Chih , Y.‐C. Chen , et al., “Additive Engineering With Triple Cations and Bifunctional Sulfamic Acid for Tin Perovskite Solar Cells Attaining a PCE Value of 12.5% Without Hysteresis,” ACS Energy Letters 7 (2022): 4436–4442, 10.1021/acsenergylett.2c02148.

[9] E. Jokar , H.‐S. Chuang , C.‐H. Kuan , et al., “Slow Passivation and Inverted Hysteresis for Hybrid Tin Perovskite Solar Cells Attaining 13.5% via Sequential Deposition,” The Journal of Physical Chemistry Letters 12 (2021): 10106–10111, 10.1021/acs.jpclett.1c03107. 34633820

[10] C.‐H. Kuan , T.‐S. Liao , S. Narra , et al., “Co‐Cation Engineering via Mixing of Acetamidinium and Rubidium in FASnI_3_ for Tin Perovskite Solar Cells to Attain 14.5% Efficiency,” The Journal of Physical Chemistry Letters 15 (2024): 7763–7769, 10.1021/acs.jpclett.4c01695. PMC11299185 39046929

[11] X. Jiang , F. Wang , Q. Wei , et al., “Ultra‐High Open‐Circuit Voltage Of Tin Perovskite Solar Cells Via An Electron Transporting Layer Design,” Nature Communications 11 (2020): 1245, 10.1038/s41467-020-15078-2. PMC7060347 32144245

[12] E. Jokar , C.‐H. Chien , A. Fathi , M. Rameez , Y.‐H. Chang , and E. W.‐G. Diau , “Slow Surface Passivation And Crystal Relaxation With Additives To Improve Device Performance And Durability For Tin‐Based Perovskite Solar Cells,” Energy & Environmental Science 11 (2018): 2353–2362, 10.1039/C8EE00956B.

[13] E. Jokar , P.‐Y. Cheng , C.‐Y. Lin , S. Narra , S. Shahbazi , and E. Wei‐Guang Diau , “Enhanced Performance and Stability of 3D/2D Tin Perovskite Solar Cells Fabricated With a Sequential Solution Deposition,” ACS Energy Letters 6 (2021): 485–492, 10.1021/acsenergylett.0c02305.

[14] E. Jokar , C. H. Chien , C. M. Tsai , A. Fathi , and E. W. G. Diau , “Robust Tin‐Based Perovskite Solar Cells With Hybrid Organic Cations to Attain Efficiency Approaching 10%,” Advanced Materials 31 (2019): 1804835, 10.1002/adma.201804835. 30411826

[15] J. Y. Park , W. Mihalyi‐Koch , C. T. Triggs , et al., “A Lead‐Free Ferroelectric 2D Dion–Jacobson Tin Iodide Perovskite,” Advanced Materials 36 (2024): 2314292, 10.1002/adma.202314292. 38684071

[16] E. W.‐G. Diau , E. Jokar , and M. Rameez , “Strategies To Improve Performance and Stability for Tin‐Based Perovskite Solar Cells,” ACS Energy Letters 4 (2019): 1930–1937, 10.1021/acsenergylett.9b01179.

[17] S. Shahbazi , M.‐Y. Li , A. Fathi , and E. W.‐G. Diau , “Realizing a Cosolvent System for Stable Tin‐Based Perovskite Solar Cells Using a Two‐Step Deposition Approach,” ACS Energy Letters 5 (2020): 2508–2511, 10.1021/acsenergylett.0c01190.

[18] X. Liu , T. Wu , X. Luo , et al., “Lead‐Free Perovskite Solar Cells With Over 10% Efficiency and Size 1 cm^2^ Enabled by Solvent–Crystallization Regulation in a Two‐Step Deposition Method,” ACS Energy Lett 7 (2021): 425–431.

[19] H. Zhou , W. Sheng , H. Rao , et al., “Reaction‐Diffusion and Crystallization Kinetics Modulation of Two‐Step Deposited Tin‐Based Perovskite Film via Reducing Atmosphere,” Angewandte Chemie 137 (2025): 202422217, 10.1002/ange.202422217. 39625462

[20] S. Wang , Y. Chen , R. Li , et al., “Superior Textured Film and Process Tolerance Enabled by Intermediate‐State Engineering for High‐Efficiency Perovskite Solar Cells,” Advanced Science 7 (2020): 1903009, 10.1002/advs.201903009. PMC7055579 32154081

[21] L. Gu , S. Wang , Y. Chen , et al., “Stable High‐Performance Perovskite Solar Cells via Passivation of the Grain Boundary and Interface,” ACS Applied Energy Materials 4 (2021): 6883–6891, 10.1021/acsaem.1c01013.

[22] C.‐H. Kuan , Y.‐A. Ko , and E. Wei‐Guang Diau , “Surface and Interfacial Passivations for FASnI_3_ Solar Cells With Co‐Cations,” ACS Energy Letters 8 (2023): 2423–2425, 10.1021/acsenergylett.3c00742.

[23] L. Lanzetta , T. Webb , N. Zibouche , et al., “Degradation Mechanism Of Hybrid Tin‐Based Perovskite Solar Cells And The Critical Role Of Tin (IV) Iodide,” Nature Communications 12 (2021): 2853, 10.1038/s41467-021-22864-z. PMC8121806 33990560

