# Supplementary material for: Synergistic Additive and Bulky Cation Engineering via a Sequential Vacuum Dry‐Dipping Process for Tin Perovskite Solar Cells
Source: Adv Sci (Weinh). 2026 Oct 6:e76969. Online ahead of print. doi: 10.1002/advs.76969 (PMC13641053; doi:10.1002/advs.76969)
Supplement: Supplementary file 1 [file ADVS-9999-e76969-s001.docx]

**Supplementary Information**

**Synergistic Additive and Bulky Cation Engineering via a Sequential Vacuum Dry-Dipping Process for Tin Perovskite Solar Cells**

Chun-Hsiao Kuan, ^‡a,b^ Xianyuan Jiang, ^‡c^ Yun-Sheng Shih, ^‡a^ Chien-Ling Ting,^a^ Po-Kai Kang,^a^ Yue Lu,^c,d^ Sung-Fu Hung,^a,b^ Hsin-Lung Chen,^e^ Shiang Lan,^a^ Guan-Ruei Chen,^f^ Jhih-Min Lin,^f^ Chun-Jen Su,^f^ Wei-Ru Wu,^f^ and Eric Wei-Guang Diau^*,a,b^

^a^Department of Applied Chemistry and Institute of Molecular Science, National Yang Ming Chiao Tung University 1001 Ta-Hseuh Rd., Hsinchu 300093, Taiwan, E-mail: [diau@nycu.edu.tw](mailto:diau@nycu.edu.tw);

^b^Center for Emergent Functional Matter Science, National Yang Ming Chiao Tung University 1001 Ta-Hseuh Rd., Hsinchu 300093, Taiwan;

^c^School of Physical Science and Technology, ShanghaiTech University, 393 Middle Huaxia Road, Pudong New Area, Shanghai 201210, China;

^d^Department of Chemistry, National University of Singapore, 3 Science Drive 3, Singapore 117543, Singapore

^e^Department of Chemical Engineering, National Tsing Hua University, No. 101, Section 2, Kuang-Fu Road, Hsinchu 30013, Taiwan;

^f^National Synchrotron Radiation Research Center, 101 Hsin-Ann Road, Hsinchu Science Park, Hsinchu 300092, Taiwan.

^‡^C.-H. Kuan, X. Jiang and Y.-S. Shih contributed equally to this work.

***Chemicals***

FAI (Greatcell materials), BATO (Greatcell materials), BDEA (Greatcell materials), PEAI (Greatcell materials), BAI (Greatcell materials), GAI (Greatcell materials), TMSO (SigmaAldrich), SnI_2_ (99.999 %, Alfa Aesar), SnF_2_ (99 %, Sigma-Aldrich), DMSO (SigmaAldrich), chlorobenzene (Sigma-Aldrich), IPA (Sigma-Aldrich), hexafluoro-2-propanol (HFP, TCI), EDAI_2_ (Greatcell materials) were used as received.

***Device fabrication***

The commercial ITO glasses (1.9×1.9 cm^2^) were first cleaned with a solution of IPA, acetone, and distilled water under sonication in a bath for 30 minutes. After this, they were further cleaned with distilled water for another 30 minutes. Prior to deposition of the HTM, PEDOT:PSS, the ITO glasses underwent a UV-ozone treatment for 15 minutes.

The tin perovskite solutions of the first step included SnI_2_ (0.8 mM) with SnF_2_ (0.16 mM), EDAI_2_ (0.008 mM) in DMSO, and those of the second step contained FAI (187.5 mg) /1. BAI (25 mg) 2. BATo (30 mg) 3. BDEA (37 mg) / CB:IPA:HFP=2:5:5 (2.5 mL/6.25 mL/6.45 mL)

The prepared SnI₂ precursor solution was drop-cast onto a glass substrate and uniformly spread across the surface using a manually controlled blade. Excess solution was absorbed with filter paper. After resting for approximately one minute, the substrate surface was blown with a nitrogen gun at a fixed angle and distance to remove residual solution. The airflow was kept uniform to avoid thickness variations caused by uneven gas pressure. The substrate was then placed under vacuum to thoroughly remove any remaining solvent, yielding an SnI₂ film.

Next, the prepared co-solution was poured into a dipping container, and the SnI₂-coated substrate from the first step was rapidly and vertically immersed for 20–30 s. The substrate was withdrawn vertically at a uniform and swift speed, laid horizontally, and immediately blown with nitrogen to remove excess solution. A second vacuum treatment was applied to ensure complete solvent removal, followed by annealing at 70 °C for 10 min to complete perovskite crystallization. Finally, an electron-transport layer (C60) with a thickness of approximately 35 nm, a hole-blocking layer (BCP) with a thickness of approximately 5 nm, and a top metal layer (Ag) with a thickness of approximately 100 nm were deposited through thermal evaporation at a pressure of approximately 5×10^-6^ Torr.

***Procedure optimization***

**The Procession construction**

Since this study represents our laboratory’s first attempt at the blade-dip coating method, establishing a complete device fabrication process and achieving efficient solar cells posed a major challenge at the initial stage. **Figure S2a** illustrates the blade-dip process. We used precursor concentration as the main means to tune film thickness. Because the initial SnI_2_ precursor film plays a decisive role in device performance, the blade–substrate gap could not be too narrow; otherwise, the deposited film became excessively thin. Conversely, excess residual solvent caused problems during N₂ quenching: the strong gas flow near the nozzle pushed solution toward the rear side of the ITO glass, creating streaks and severe film nonuniformity that degraded device efficiency. By inserting filter paper under the ITO to absorb excess solution and maintaining a fixed N₂-gun position, we obtained improved films (**Figure S2b**).

The second step, a dip-coating conversion of the SnI_2_ film to perovskite, was carried out by vertical immersion rather than horizontal dipping. This approach reduced FAI solution consumption, minimized solvent leakage, and lessened glovebox contamination, which can interfere with crystallization. Importantly, the immersion must be instantaneous to avoid uneven crystallization between the upper and lower regions of the film. Since no prior reports exist for tin-based systems, immersion time first had to be optimized. **Figures S3–S4** show that perovskite films immersed for 20–30 s exhibit the deepest brown color, best crystallinity, and uniform morphology, whereas longer immersion (40–180 s) resulted in transparency, grain breakage, higher grain-boundary density, and thus greater defect density. AFM analysis (**Figure S5**) confirmed that surface roughness was lowest at 20 s, with smoother topography and superior uniformity. Shorter (10 s) immersion left unreacted SnI₂, while longer times led to sparse grains and rougher films. Repeated trials confirmed that 20 s immersion produced the best device efficiency, yielding an initial PCE of 4.1% (**Figure S6**).

After optimizing immersion, we identified a critical efficiency-limiting factor: pinholes on the film surface (**Figure S7**). These originated from two sources. First, residual DMSO in the SnI₂ film could not be fully removed by N₂ blowing, unlike in spin-coating processes where most solvent is flung away. Excess DMSO caused micro-voids upon subsequent conversion. Attempts using heating or solvent exchange damaged the precursor due to Sn oxidation, but vacuum treatment successfully removed DMSO, increasing device current and fill factor, and raising PCE to 5.4% (**Figure S8-S9**). However, manual blade-coating introduced unavoidable nonuniformity, leaving irregular streaks. Second, SEM/AFM characterization (**Figure S10**) revealed nanoscale pinholes in the converted perovskite, attributed to excessive FAI solution vapor inside the glovebox that etched the film during annealing. Reducing device batch size and applying post-dip vacuum alleviated these issues, underscoring the importance of vacuum treatment in both steps.

Further characterization confirmed the strong correlation between immersion time and film quality. XRD patterns (**Figures S11–S12**) showed no phase shift in the perovskite lattice with immersion time, but the (100) peak intensity peaked at 20 s, indicating optimal crystallinity. Steady-state PL (**Figures S13–S14**) likewise showed strongest emission at 20 s with no spectral shift (~870 nm), while longer immersion produced broader, distorted peaks reflecting poor crystallinity. UV–Vis absorption (**Figure S16**) revealed the strongest absorption at 20 s with an optical bandgap of 1.82 eV (from Tauc analysis). UPS (**Figure S15**) determined valence and conduction band energies of −5.22 eV and −3.40 eV, respectively.

In summary, immersion time of 20–30 s is optimal for blade-dip coating of tin perovskites, with 20 s providing the best crystallinity, morphology, carrier dynamics, and device performance. All subsequent additive engineering experiments were therefore carried out using 20 s immersion to identify the most effective formulations.

**Adding GAI**

With the blade–dip coating method showing PCE = 5.1%, we next explored ways to further improve efficiency. Guanidinium iodide (GAI), previously employed as an additive in our two-step spin-coating process, was introduced into the blade–dip approach. GA⁺ is known to passivate and heal defects in perovskite films. By incorporating 80, 90, and 100 mg of GAI into the FAI solution, we observed a marked improvement in device performance relative to pristine FASnI_3_. In particular, the device with 90 mg GAI achieved a champion PCE of 7.1%, with *V*oc exceeding 0.6 V and notable enhancement of the fill factor (**Figure S17**).

SEM analysis (**Figure S18**) revealed that compared with spin-coated references, the blade–dip method yielded more uniform crystallites, especially at the optimized GAI concentration. At 90 mg, perovskite grains were larger and more compact, while excessive GAI (100 mg) accelerated crystallization, leading to pinholes and pronounced grain boundaries, thereby reducing device performance. AFM results (**Figure S19**) showed a minimum roughness of 26.9 nm at 90 mg GAI, while higher concentrations again increased roughness and surface undulation.

XRD (**Figure S20**) confirmed no shift in the perovskite (100) peak, but the relative intensity at 14° (perovskite) vs. 34° (ITO) was highest at 90 mg, indicating superior crystallinity. PL spectra (**Figure S21**) showed a progressive blue shift with increasing GAI, attributed to lattice distortion caused by the larger GA⁺ cation (~2.78 Å vs. FA⁺ ~2.5 Å). UV–Vis spectra (**Figure S22**) further demonstrated enhanced absorption at 830 nm for 90 mg GAI, while higher loading reduced absorption strength. Tauc analysis (**Figure S23**) showed a widening bandgap with GAI addition, consistent with VB modulation observed in UPS (**Figure S24**). Combined energy-level diagrams (**Figure S25**) indicated that 90 mg GAI yielded the most favorable alignment.

XPS analysis (**Figure S26 and Table S3**) further showed that 90 mg GAI minimized Sn^4+^ defect formation, whereas excessive loading increased Sn oxidation due to accelerated crystallization. Finally, devices based on FA₀.₇GA₀.₃SnI₃–1% EDAI₂ absorbers in inverted architecture (ITO / PEDOT:PSS / absorber / C₆₀ / BCP / Ag) were systematically compared. Box plots of photovoltaic parameters over 15 devices per condition (**Figures S27**) demonstrated reproducible efficiency improvements, with the highest median PCE at 90 mg GAI. Beyond this loading, device metrics deteriorated, confirming 90 mg as the optimal additive concentration.

In summary, introducing GAI into the blade–dip process significantly improved device performance, raising efficiency from 5.1% to 7.1% by defect passivation, crystallinity enhancement, and uniform cation incorporation.

**Incorporation of SnF_2_ into Blade–Dip Coated Devices**

Since the blade–dip coating method was initially adapted from our laboratory’s two-step spin-coating recipe, the precursor formulation also employed fixed ratios of 20 mol% SnF_2_ and 1 mol% EDAI_2_. After introducing Guanidinium iodide (GAI), we observed that—despite identical precursor ratios—the passivation effect and device efficiency varied greatly depending on the fabrication method. Therefore, we systematically optimized the SnF_2_ content. Adjusting SnI_2_ or EDAI_2_ concentrations yielded negligible changes, whereas increasing SnF_2_ concentration produced a significant improvement in device efficiency. As shown in **Figure S28**, raising the SnF_2_ content to 30 mol% increased the PCE to 8.3%, with *V*oc and fill factor reaching 0.70 V and 72.5%, respectively. Beyond this level, efficiency declined due to phase separation and defect formation caused by excessive SnF_2_. The detailed photovoltaic parameters for different SnF_2_ ratios are listed in **Table S5**. SEM images (**Figure S29**) showed no major differences between 20% and 30% SnF_2_ conditions; however, at 40% SnF_2_, large white particles appeared, likely corresponding to SnI_2_ precipitates, which degraded efficiency. AFM analysis (**Figure S30**) revealed that films with 30% SnF_2_ exhibited reduced roughness (17.6 nm) and more uniform crystallite height distribution. Cross-sectional SEM (**Figure S31**) confirmed this trend: (a) pristine FASnI_3_ with 20% SnF_2_ showed porous and uneven films; (b) adding 90 mg GAI reduced voids and improved uniformity; (c) combining 90 mg GAI with 30% SnF_2_ produced the flattest morphology and the thickest, most homogeneous film (thickness ~240 nm). At 40% SnF_2_, however, the film thickness decreased, confirming the detrimental impact of excess SnF_2_. XRD patterns (**Figure S32**) demonstrated enhanced crystallinity at 30% SnF_2_ under 90 mg GAI. PL spectra (**Figure S33**) showed a red shift with increasing SnF_2_ content, attributed to lattice stress and bandgap narrowing.

UV–Vis absorption spectra (**Figure S34**) confirmed stronger absorption at 30% SnF_2_. XPS analysis (**Figure S35** and **Table S6**) showed that 30% SnF_2_ minimized Sn⁴⁺ defect formation, whereas higher concentrations increased oxidation due to accelerated crystallization. Reproducibility tests on FA₀.₇GA₀.₃SnI₃–1% EDAI₂ absorbers confirmed that 30% SnF_2_ yielded the highest efficiency (8.26%) with improved *V*oc and FF (**Figure S36**, **Table S4**). IPCE spectra (**Figure S37**) suggested further optimization is required to increase photocurrent. *J–V* curves under forward and reverse scans (**Figure S38**) showed negligible hysteresis (<1% PCE deviation) for all SnF_2_ levels. Electrochemical impedance spectroscopy (EIS, **Figures S39–S40**) revealed the highest recombination resistance (R_rec_) for FA₀.₇GA₀.₃SnI₃–30% SnF₂, indicating the most efficient charge separation.

In summary, increasing the SnF_2_ ratio to 30% in the blade–dip process synergistically improved morphology, crystallinity, carrier lifetime, and stability, raising efficiency from 5–7% (with GAI) to a new high value of 8.3%. Excess SnF_2_, however, induced phase separation and performance loss, underscoring the need for careful balance.

**Adding PEAI**

While 3D tin perovskites exhibit excellent optoelectronic properties, they are highly unstable to air and moisture. Introducing bulky organic cations such as phenylethylammonium (PEA⁺) can form 2D or mixed-dimensional (quasi-2D/3D) structures, suppress oxidation, and improve environmental stability. However, excessive 2D content often introduces insulating organic layers that impede carrier transport, leading to discontinuous pathways and reduced photovoltaic performance. Thus, combining scalable blade–dip coating with mixed-dimensional design offers a promising strategy to achieve high stability, reproducibility, and efficiency in large-area tin perovskite modules.

PEAI is a commonly used bulky cation in Ruddlesden–Popper (RP) structures. Devices fabricated with varying PEAI concentrations (relative to FAI) showed the best efficiency of 6.3% at 10 mol% PEAI (**Table S7, Figure S41**). Although *V*oc remained nearly unchanged across conditions, both *J*sc and FF improved markedly. Therefore, 10 mol% PEAI was selected as the baseline for further study. SEM images (**Figure S42**) revealed strong dependence of film quality on PEAI concentration. At 5%, films still showed noticeable voids; at 10%, the films became compact, with improved grain fusion, fewer defects, and smoother morphology; whereas 15% PEAI produced quasi-2D layered features and reintroduced voids. AFM analysis (**Figure S43)** confirmed the lowest roughness at 10% PEAI (RMS = 21.6 nm), consistent with denser films and improved interfacial contact. XRD patterns **(Figures S44)** showed enhanced diffraction intensity with PEAI, indicating improved crystallinity. SIMS results further confirmed that PEA⁺ incorporated into the lattice, occupying A-site vacancies. At 15% PEAI, diffraction peaks broadened, reflecting poorer crystallinity and higher defect density. UV–Vis absorption spectra (**Figure S45**) showed stronger absorption at 10% PEAI, while both lower and higher concentrations reduced absorption intensity. PL spectra (**Figure S46**) exhibited similar emission positions (~880 nm), but the PL intensity was highest at 10% PEAI, indicating fewer defects and lower non-radiative recombination losses. XPS analysis (**Table S8, Figure S47**) quantified the Sn²⁺/Sn⁴⁺ ratio under different PEAI loadings. At 10% PEAI, the Sn⁴⁺ fraction was lowest, confirming suppressed oxidation and enhanced structural stability. Both under-doping and over-doping increased Sn⁴⁺ content, likely due to incomplete passivation and higher defect density that allowed oxygen/moisture ingress.

In summary, incorporating 10 mol% PEAI into blade–dip coated tin perovskites optimized morphology, reduced roughness, improved crystallinity, and suppressed oxidation, leading to the best balance between efficiency (6.3%), defect passivation, and stability.

**Incorporation of TMSO**

Although adding PEAI improved device performance, the enhancement was modest, likely due to residual defects and suboptimal crystallinity. To further boost efficiency, we introduced trimethylsulfoxide (TMSO) during the first step to improve film quality. Previous studies in lead-based perovskites reported that TMSO forms stable intermediate states, promotes large-grain growth, and reduces defects. However, its application in tin-based systems has not yet been reported. Based on analogous mechanisms, TMSO may coordinate with under-coordinated Sn²⁺, suppress oxidation, facilitate homogeneous nucleation, and improve crystal growth. These effects should yield dense, uniform films with fewer defects, thereby enhancing charge extraction and reducing interfacial recombination.

We investigated various TMSO loadings in the FASnI_3_ system, with results summarized in **Table S9 and Figure S48**. Adding 15 μL TMSO produced the most significant improvement: *J*sc increased from 15 mA cm⁻² to 19 mA cm⁻², FF improved to 72%, and the PCE reached 8.33%. This suggests that film defects were minimized under this condition, and TMSO played a similar beneficial role in tin perovskites as in lead systems. Consequently, 15 μL was selected as the optimal concentration for subsequent experiments. SEM images (**Figure S49**) revealed clear differences across TMSO concentrations. At 5 μL and 25 μL, films contained numerous pinholes, while 15 μL yielded the most compact morphology with reduced voids. Cross-sectional SEM (**Figure S50**) further confirmed that films without TMSO were rough and uneven, while those with 15 μL exhibited smooth surfaces, uniform thickness (~200 nm), and dense packing. AFM analysis (**Figure S51**) quantified this improvement, with the lowest RMS roughness (19.9 nm) achieved at 15 μL, confirming enhanced film uniformity. XRD patterns (**Figure S52**) showed no peak shift upon TMSO addition, indicating no structural distortion. However, diffraction peak intensity increased at 15 μL, confirming improved crystallinity and the absence of residual SnI_2_. Thus, TMSO’s role was to regulate nucleation and growth dynamics rather than to enter the lattice itself. The optimal combination of 10% PEAI and 15 μL TMSO produced the smoothest and most crystalline films. UV–Vis absorption spectra (**Figures S53–S54**) revealed stronger absorption at 15 μL TMSO, consistent with improved crystallinity and film density. Excess or insufficient TMSO led to weaker absorption due to increased defects and poorer morphology. PL spectra (**Figures S55-56**) showed unchanged emission peak positions (~880 nm), but the intensity increased markedly with 15 μL TMSO, reflecting reduced non-radiative recombination and fewer defects. XPS analysis (**Table S10, Figure S57**) demonstrated that 15 μL TMSO minimized the fraction of oxidized Sn⁴⁺, thereby suppressing defect-related degradation. At lower or higher concentrations, this beneficial effect was diminished: insufficient TMSO provided limited defect passivation, while excessive amounts interfered with crystal growth, introducing more local defects and weakening oxidation suppression.

In summary, adding 15 μL TMSO during blade–dip coating significantly enhanced film morphology, reduced roughness (RMS = 19.9 nm), improved crystallinity, increased *J*sc and FF, and suppressed Sn²⁺ oxidation. As a result, device efficiency reached 8.33%, demonstrating that controlled TMSO incorporation is an effective strategy to improve tin-based perovskite solar cells.

**Incorporation of Different Large Organic Cations**

Based on the optimized conditions identified in previous sections—namely, 10 mol% large organic cation in the second step and 15 μL TMSO—we further investigated the effect of replacing PEAI with other bulky organic cations mentioned in the main text.

At the optimum 10 mol% loading, TsO^−^ forms only a sub-nanometer passivation skin (consistent with its sharp localization in Figure 3e) that behaves as a tunneling-friendly defect-healing layer. When the BATo content is raised to 15–20 mol%, however, the device performance clearly deteriorates (**Figure S58**; revised Supplementary **Figure S69**): the FF drops from 72.2% to <60%, JSC decreases by ≈15%, confirming that beyond the optimum concentration the insulating TsO− accumulation acts as a parasitic series resistance and extraction barrier.

**Characterization of films and devices**

*J-V* curves were obtained at a scan rate of 0.07 V/s using a SAN-EI solar simulator (model XES-40S1) under standard one sun illumination (AM 1.5G spectrum, 100 mW/cm^2^), with calibration against a standard silicon reference cell (Oriel, model PN 91150V, VLSI standard). Both forward and reverse scans (from 0 V to *V*_OC_ and from *V*_OC_ to 0 V, respectively) were carried out in ambient air conditions, and the metal mask used had an area of 0.1 cm^2^. The Incident Photon-Electron Conversion Efficiency (IPCE) spectra were measured using a system consisting of a Xe lamp (A-1010, PTi, 150 W) and a monochromator (PTi), followed by recording the IPCE spectra after calibration with a standard Si photodiode (S1337-1012BQ, Hamamatsu). XRD patterns were obtained using a commercial diffractometer (Bruker D8-Advance) with Cu Kα radiation. Morphological features of the samples were recorded using a field-emission SEM (Hitachi SU8010) and an atomic-force microscope (AFM, VT SPM, SII Nanotechnology Lnc.), respectively. The samples were encapsulated with glasses using UV glue (NOA 68, Norland Products). UV-visible absorption spectra of the samples were recorded using a UV-visible spectrophotometer (V780, Jasco). The photoluminescence (PL) spectra were recorded by exciting the samples with a CW diode laser (450 nm, MDL-III-450-100 mW; power supply: PSU-III-FDA) and detecting the emission spectra between 550-1100 nm on scanning the grating of a spectrometer (Dongwoo, DM150i, 600 groves blaze: 750 nm) using a TE-cooled Si-photodiode (Sciencetech Inc. S-025-TE2-H; power supply: PS/TC-1). Electrochemical impedance spectroscopy (EIS) measurements were performed in the dark with an AC amplitude of 10 mV spanning a frequency range of 1 MHz to 1 Hz, using DC bias equal to open circuit voltage.

X-ray photoelectron spectra (XPS) and ultraviolet photoelectron spectra (UPS) were also collected using a synchrotron source at various excitation energies at Taiwan Light Source beamline 24A1 at the National Synchrotron Radiation Research Center (NSRRC). XAS (XANES and EXAFS) spectra of Sn were performed in fluorescence mode on the hard X-ray spectrometer of TLS 01C1 beam lines in NSRRC, Taiwan. The corresponding EXAFS data were analyzed through the standard procedures using the Ifeffit package. The XANES spectra of N and F were measured at the Taiwan photon source (TPS) 45A2 beamlines with a soft X-ray spectrometer.

**DFT calculation**

The construction of the atomic models and their visualization were performed using the Device Studio program developed by Hongzhiwei Technology (Shanghai) Co. Ltd. The electronic structure calculations were performed using the density functional theory (DFT) method based on the DS-PAW software package.^[1]^ The Perdew-Burke-Ernzerhof (PBE) functional under the generalized gradient approximation (GGA) was employed to describe the exchange correlation interaction.^[2]^ The plane-wave basis set was truncated at 500 eV. A vacuum layer of 15 Å was introduced in all three directions (x, y, and z) to simulate the environment of the molecule in a vacuum. The convergence criteria for the electronic self-consistent energy were set to 10^-6^ eV, with the ionic step forces converged to 0.01 eV/Å during the structure optimization.

The bonding energy (*E_b_*) is defined as:

*E_b_ = E_tot_ – E_1_ – E_2_*

where *E_tot_* is the energy of the two molecules when they are combined, and *E_1_* and *E_2_* represent the energies of the two molecules when they exist independently, respectively.

Using the same protocol as our BATo–FA–SnI_2_ calculations (PBE/GGA, 500 eV plane-wave cutoff, 15 Å vacuum), we computed the binding energy E_b_ of Sn^2+^ (and SnI_2_) with TMSO, DMSO and DMF. The optimized geometries are shown in Response **Figure R2** (revised Supplementary **Figure S61**) and the values are summarized in revised Supplementary **Table S2**:

The expected ordering E_b_(TMSO) > E_b_(DMSO) > E_b_(DMF) is independently supported by three established results, all of which can be cited directly:

(i) On the calorimetric BF_3_-affinity Lewis-basicity scale, TMSO (108.1 kJ mol^−1^) is a stronger Lewis base than DMSO (105.3 kJ mol^−1^) and DMF (≈95 kJ mol^−1^); the cyclic five-membered sulfoxide ring raises the electron density on the S=O oxygen and tightens the dative bond to Sn^2+^.^[3]^

(ii) The S=O stretching mode red-shifts by ≈40 cm^−1^ upon TMSO–PbI_2_ adduct formation, markedly larger than the ≈25 cm^−1^ shift for DMSO–PbI_2_, directly evidencing a stronger metal–O dative bond; the TMSO–PbI_2_ adduct also shows a higher decomposition temperature.^[4]^

(iii) Recent DFT work shows that SnI_2_ binds DMSO more strongly than PbI_2_ does at low coordination numbers (the SnI_2_·3DMSO cohesive energy is −1.920 eV), confirming that sulfoxide ligands coordinate Sn^2+^ effectively.^[5]^

**TCSPC measurements**

Time-Correlated Single Photon Counting (TCSPC) measurements were conducted utilizing a TCSPC setup (Fluotime 200, Picoquant brand), which used a laser with an excitation wavelength of 635 nanometers. These measurements focused on capturing transient decay profiles at the peak wavelengths observed in the PL spectra. Throughout these experiments, the laser operated at a consistent repetition rate of 25 MHz, and its pulse energy was maintained at 4 microjoules per square centimeter. The collected transient PL profiles were then meticulously analyzed and interpreted using a bi- or tri-exponential function model to understand their decay characteristics.

**TOF-SIMS**

Depth profiles using Time-of-Flight Secondary Ion Mass Spectrometry (TOF-SIMS) were acquired with a PHI TRIFT V nanoTOF system (manufactured by ULVAC-PHI, Japan), utilizing a dual beam technique that combines slicing and viewing. The data collection process involved the use of a pulsed C60^+^ primary ion beam, set at an energy of 20 kV, with a pulse frequency of 8200 Hertz and a pulse duration of 15 ns, which was instrumental in generating secondary-ion signals. This primary ion beam maintained a current of 0.15 nA (DC). The targeted area for analysis was defined as 50 μm by 50 μm. To accelerate the secondary ions, a 3 kV bias was applied to the sample. During the data acquisition process, both a 10 V electron flood and a 10 V Ar^+^ flood were used to neutralize any surface charge. In order to compensate for variations in the primary ion signal, all secondary-ion signals within the depth profiles were normalized against the total ion signals. For the sputtering phase, aimed at removing material from the surface, a 1 kV Ar^+^ ion beam with a current of 100 nA (DC) was utilized. The raster area designated for this Ar^+^ sputtering beam was 2 mm by 2 mm.

**Grazing-incidence Wide-angle X-ray Scattering (GIWAXS) measurements**

The end station of beamline TPS 25A1 at NSRRC, Taiwan, was used for GIWAXS measurements. For the sample structure analysis using grazing incident X-ray diffraction (GIXRD), the X-ray photon energy of the incident beam was 12.37 keV and the measurements were conducted at TPS 25A1, NSRRC. The beam sizes at TPS 25A1 were 5 μm by 5 μm and the q-range of the measurements with a sample-to-detector distance of 85 mm was 0.1 ~ 5 Å^-1^. With an incident angle at 0.05 degree, the diffraction from the film was enhanced and the two-dimensional diffraction patterns were recorded by a single-photon counting detector, Eiger X 1M, with a pixel size of 75 μm.

**In-situ GIWAXS measurements**

In-situ GIWAXS experiments for the perovskite blade-diping process were conducted at TLS 23A wide-angle X-ray scattering (SWAXS) beamline at NSRRC, Taiwan.

**Revised Supplementary Note 1 - Scale-up and vacuum-drying parameters.**

– Wet-film thickness, set by the blade gap (≈180–220 µm) and translation speed (≈15–25 mm s−1); a faster speed thins the wet film and avoids meniscus instability.

– N_2_-quenching uniformity, achieved with an air-knife nozzle traversing the substrate at constant height and speed to homogenize residual-DMSO removal and prevent streaks/pinholes.

– Vacuum pump-down profile (the most critical factor): a controlled ramp of ≈10 s from 1 atm to <5 Pa. A slower pump-down (>30 s) causes solute pinning and coffee-ring defects; a faster one (<5 s) causes bubble nucleation and delamination. The optimized ramp gives film-thickness variation <±5% across the full 5 × 5 cm^2^ area.

**Revised Supplementary Note 2 - KPFM Analysis detail**

To investigate the effect of the bulky cations (PEAI and BATo) on the nanoscale surface electronic landscape of the perovskite films, Kelvin Probe Force Microscopy (KPFM) was performed alongside topography measurements. The surface potential maps were acquired in amplitude-modulation (AM) mode using a commercial atomic force microscope equipped with a conductive Pt-coated silicon probe. To prevent any light-induced photovoltage effects or ambient moisture degradation, all scans were conducted over a 5 × 5 µm² area under a controlled N₂ environment in the dark. In KPFM, the applied DC voltage nullifies the contact potential difference (V_CPD_) between the conductive tip and the sample surface, providing a direct mapping of the local surface potential. For pristine perovskite films, the under-coordinated ions and charged defects typically segregate at the grain boundaries (GBs), leading to a significant potential contrast (ΔV_grain-GB_) between the grain interiors and the GBs. This nanoscale potential fluctuation creates localized band-bending wells that act as non-radiative recombination centers. Upon surface treatment, the observed V_CPD_ maps directly reflect the efficacy of defect passivation. A decrease in the potential roughness [σ(V_CPD_)] and the grain/GB contrast (ΔV_grain-GB_) indicates a more homogeneous surface electronic state. In our study, the BATo-modified film exhibited the most minimized σ(V_CPD_) and ΔV_grain-GB_ compared to the pristine and PEAI-modified samples. This potential homogenization suggests that the synergistic interaction of the BATo molecule—where the TsO⁻ group coordinates with under-coordinated Sn²⁺ traps via S=O → Sn²⁺ dative bonds and the BA⁺ group restores local A-site vacancies—effectively neutralizes the charged defects at the GBs. Consequently, this flattened potential landscape suppresses charge carrier trapping and non-radiative recombination at the interface, consistently supporting the enhanced charge carrier lifetime and device V_OC_ discussed in the main text.

**Revised Supplementary Note 3 - Stability test protocols**

To systematically evaluate the reliability of the fabricated perovskite solar cells, stability tests were conducted in accordance with the established ISOS protocols. The detailed experimental conditions for the shelf-storage and continuous operational stability tests are provided below:

- **Shelf-Storage Stability (ISOS-D-1I Protocol)** To assess the long-term environmental durability, the shelf-storage stability of the encapsulated devices was monitored over a total duration of 2500 h. The devices were stored in the dark inside a nitrogen-filled glovebox with strictly controlled atmospheric conditions (O2 < 0.1 ppm and H2O < 0.1 ppm) at a constant temperature of 25 ± 2 °C. To track the degradation profile, the devices were periodically removed from the glovebox to undergo standard current density–voltage (J–V) measurements under ambient conditions, after which they were promptly returned to the storage environment.
- **Continuous Operational Stability (ISOS-L-1I Protocol)** The operational stability under continuous illumination was evaluated via maximum power point tracking (MPPT). The encapsulated devices were tested in an inert atmosphere (O2 < 1 ppm and H2O < 1 ppm) at a stabilized temperature of 25 ± 2 °C. The devices were subjected to continuous simulated AM 1.5G illumination (100 mW cm−2) for a total duration of 5 h. The maximum power point was continuously tracked using a perturb-and-observe algorithm with a voltage step of 5 mV and an update interval of 1 s.

**Figure S1.** Corresponding 1D intensity profiles extracted from the 2D patterns. The VCD film shows broad and diffuse scattering, characteristic of an amorphous or poorly ordered structure. In contrast, the annealed film exhibits sharp diffraction spots in the 2D patterns and well-defined Bragg peaks in the 1D profile, indicating significant crystallization induced by thermal annealing.

**Figure S2.** (a) Relatively uniform SnI₂ precursor film prepared by blade-coating. (b) Surface streaks caused by nitrogen blowing due to residual solvent.

**Figure S3.** Film appearance at different immersion times.

**Figure S4.** Surface SEM images of perovskite films with different immersion times.

**Figure S5.** AFM images of perovskite films obtained at different immersion times.

**Figure S6.** Immersion time variation chart of the same-batch experiment.

**Figure S7.** Image of the placed holes.

**Figure S8.** Improvement of the device after vacuum treatment.

**Figure S9.** Efficiency improvement under vacuum treatment at the same immersion time.

**Figure S10.** SEM measurement image (micropore image).

**Figure S11.** X-ray patterns with varying immersion times.

**Figure S12.** Ratio plot of ITO and perovskite signals with different dipping time

**Figure S13.** Photoluminescence spectra with varying immersion times.

**Figure S14.** Normalized photoluminescence spectra with varying immersion times.

**Figure S15.** UPS spectrum after 20 s immersion.

**Figure S16.** UV–Vis spectra with varying immersion times.

**Figure S17.** Efficiency plots with different GAI ratios.

**Figure S18.** (a) SEM image of 90 mg GAI (spin–dip coating); (b–d) SEM images of different GAI ratios (blade–dip coating).

**Figure S19.** AFM images with different GAI ratios.

**Figure S20.** XRD patterns with different GAI ratios.

**Figure S21.** PL spectra with different GAI ratios.

**Figure S22.** UV–Vis spectra with different GAI ratios.

**Figure S23.** UV–Vis spectra with different GAI ratios.

**Figure S24.** UPS spectra with different GAI ratios.

**Figure S25.** Energy level diagrams with varying GAI contents.

**Figure S26.** XPS spectra with different GAI ratios.

**Figure S27.** Photovoltaic parameters (Jsc, Voc, FF, PCE) with varying GAI contents and SnF₂ optimization.

**Figure S28.** Efficiency plots with varying SnF₂ concentrations.

**Figure S29.** SEM images with different SnF₂ concentrations. *Figure S29a is identical to Fig. S18c, as the optimized condition (90 GAI) from the previous stage serves as the baseline control (20 SnF_2_) for this step.*

**Figure S30.** SEM images with different SnF₂ concentrations. *Figure S30a is identical to Fig. S19b, as the optimized condition (90 GAI) from the previous stage serves as the baseline control (20 SnF2) for this step.*

**Figure S31.** Cross-sectional SEM comparison of perovskite films with the same ratio.

**Figure S32.** XRD patterns with varying SnF₂ concentrations.

**Figure S33.** PL spectra with varying SnF₂ concentrations.

**Figure S34.** UV–Vis spectra with varying SnF₂ concentrations.

**Figure S35.** XPS spectra with varying SnF₂ concentrations.

**Figure S36.** Photovoltaic parameters (Jsc, Voc, FF, PCE) with different SnF₂ contents.

**Figure S37.** IPCE spectra of pure FASnI₃, FA₀.₇GA₀.₃SnI₃, and FA₀.₇GA₀.₃SnI₃–30% SnF₂.

**Figure S38.** (a) 20%, (b) 30%, and (c) 40% SnF₂ forward and reverse scan J–V curves.

**Figure S39.** Equivalent circuit diagram of the device.

**Figure S40**. EIS spectra under different conditions.

**Figure S41**. I–V curves with different PEAI concentrations.

Figure S42 SEM images of perovskite films with varying PEAI additive amounts

**Figure S43.** AFM images with varying PEAI contents

**Figure S44**. XRD patterns with varying PEAI contents.

**Figure S45.** Absorption spectra with varying PEAI contents.

**Figure S46.** Photoluminescence spectra with varying PEAI contents.

Figure S47. XPS spectra with varying (a) 5%, (b) 10% and (c) 15% PEAI contents.

Figure S48. I–V curves with different TMSO concentrations.

**Figure S49.** SEM images with varying TMSO contents. *Figure S49 (0ul TMSO) is identical to Fig. S42 (10%PEAI), as the optimized condition (10%PEAI) from the previous stage serves as the baseline control (0ul TMSO) for this step.*

**Figure S50.** Cross-sectional SEM images before and after TMSO addition

**Figure S51.** AFM images with varying TMSO contents. *Figure S51 (0ul TMSO) is identical to Fig. S43 (10%PEAI), as the optimized condition (10%PEAI) from the previous stage serves as the baseline control (0ul TMSO) for this step.*

**Figure S52.** XRD patterns with varying TMSO contents.

**Figure S53**. Absorption spectra before and after TMSO addition.

**Figure S54.** Absorption spectra with varying TMSO contents.

**Figure S55.** Photoluminescence spectra before and after TMSO addition.

**Figure S56.** Photoluminescence spectra with varying TMSO contents.

**Figure S57.** XPS spectra with varying TMSO contents.

**Revise Figure S58.** J–V curves of perovskite solar cells with varying BATo concentrations (10%, 15%, and 20%). The 10% loading yields the optimal performance. Excessive loading (15% and 20%) severely degrades both Js and FF, as the accumulation of bulky organic molecules creates an insulating barrier that hinders charge extraction.

**Revise Figure S59.** **(a)** FTIR spectra of pure BATo powder and the BATo-modified film on PEDOT:PSS (locally magnified). The S=O peak red-shift (from 1181.6 to 1179.5 cm⁻¹) confirms the chemical affinity between TsO⁻ and the underlying HTL. **(b)** Top-view SEM and sulfur EDS mapping of the mechanically delaminated bottom surface. The uniform sulfur signals directly visualize the strong accumulation of TsO⁻ at the buried interface.

**Figure S60.** Low angle XRD (2θ = 2°–15°) patterns of tin perovskite film incorporating with BATo cations.

**Figure S61.** (a) Distributions of the charge density from DFT and (b) binding energies of SnI_2_–DMF, SnI_2_–DMSO, and SnI_2_–TMSO.

**Figure S62.** SEM images under different large organic cation conditions.

**Figure S63.** AFM images under different large organic cation conditions. *Figure S63 (PEAI) is identical to Fig. S51 (15ul TMSO), as the optimized condition (15ul TMSO) from the previous stage serves as the baseline control (PEAI) for this step.*

**Figure S64.** Enlarged XRD main peaks under different large organic cation conditions.

**Figure S65**. Tauc plot results under different large organic cation conditions.

**Figure S66**. UPS fitting results under different large organic cation conditions.

**Figure S67.** KPFM result for pristine (FAI-only), PEAI-modified and BATo-modified perovskite films

**Figure S68.** Comparison of charge carrier dynamics for perovskite treated with different passivation molecules (PEAI, BDEA, BAI, and BATo). The time-correlated single-photon counting (TCSPC) photoluminescence lifetime (left axis) of the films and the effective recombination time (τ_rec_, right axis) of the full devices are presented. The BATo-modified sample exhibits the longest lifetimes at both the microscopic (film) and macroscopic (device) levels, unambiguously demonstrating its superior effectiveness in suppressing non-radiative recombination.

Figure S69. XPS spectra under different large organic cation conditions.

**Figure S70.** Box plots of device efficiency parameters under different large organic cation conditions.

**Figure S71.** MPPT profiles of devices with BATo large organic cation addition.

**Figure S72.** (a) Photograph of the large-area 5 × 5 cm^2^ perovskite films. (b) 2D spatial mapping of the power conversion efficiency (PCE) for a 4 × 4 array of sub-cells (0.1 cm² each) diced from a 5 × 5 cm² perovskite film. The heatmap clearly demonstrates excellent macroscopic uniformity, with ≈ 88% of the devices exhibiting a PCE > 9% and the champion cell reaching 10%.

**Figure S73.** (a) Photograph of the large-area device with an active area of ≈ 2.6 cm². (b) The corresponding J–V curve, showing a PCE of 8.46 %. This demonstrates the excellent macroscopic uniformity and scalability of the blade-coated films.

Table S1. TCSPC parameters with varying immersion times.

| **TCSPC** | **10s** | **20s** | **30s** | **40s** | **50s** |
| --- | --- | --- | --- | --- | --- |
| t1(A1) | 0.40(85%) | 2.2(72%) | 0.47(49%) | 0.18(25%) | 0.13(79%) |
| t2(A2) | 1.08(15%) | 4.2(28%) | 1.22(51%) | 0.06(75%) | 0.33(21%) |
| Life time **(ns)** | 0.6 | 3.1 | 1.01 | 1.14 | 0.2 |

Table S2. Measurement parameters with different GAI ratios.

| **GAI(mg)** | **PCE(%)** | **Voc(V)** | **Jsc(mA/cm^2^)** | **Fill Factor (%)** |
| --- | --- | --- | --- | --- |
| 90(x=0.30) | 7.1 | 0.631 | 15.60 | 72 |
| 80(x=0.28) | 6.1 | 0.615 | 15.725 | 62.97 |
| 100(x=0.33) | 5.68 | 0.608 | 13.2 | 70.8 |

Table S3. TCSPC parameters with varying GAI concentrations.

|  | 20SnF2 80GAI | 20SnF2 90GAI | 20SnF2 100GAI |
| --- | --- | --- | --- |
| T1(A1) | 3.4(67%) | 3.1%(65%) | 14.4(12%) |
| T2(A2) | 12.7(33%) | 15.4(35%) | 1.79(88%) |
| Life time(ns) | 9.4 | 11.9 | 8.4 |

Table S4. Sn²⁺ and Sn⁴⁺ ratios at different SnF₂ proportions.

|  | 80mg | 90mg | 100 mg |
| --- | --- | --- | --- |
| Sn2+ | 92.5% | 95.4% | 83.8% |
| Sn4+ | 7.5% | 4.96% | 16.2% |

Table S5. Measurement parameters with different SnF₂ ratios.

| SnF2 | PCE(%) | Voc(V) | Jsc(Ma/cm^2^) | Fill Factor(%) |
| --- | --- | --- | --- | --- |
| 30% | 8.15±0.010 | 0.701±0.002 | 16.35±0.44 | 71.1±0.65 |
| 20% | 6.9±0.14 | 0.638±0.006 | 16.41±1.02 | 64.6±0.65 |
| 40% | 7.6±0.12 | 0.685±0.053 | 17.10±0.31 | 66.2±1.02 |

Table S6. Sn²⁺ and Sn⁴⁺ ratios at different SnF₂ proportions.

|  | 20% SnF2 | 30% SnF2 | 40% SnF2 |
| --- | --- | --- | --- |
| Sn2+ | 95.4% | 97.43% | 96.38% |
| Sn4+ | 4.96% | 2.57% | 3.62% |

Table S7. Efficiency data with different PEAI concentrations.

| PEAI conc. | Voc (V) | Jsc (mA cm-2) | FF | PCE (%) |
| --- | --- | --- | --- | --- |
| 0% (FAI) | 0.591 | 13.54 | 64.31 | 5.15 |
| 5% | 0.574 | 14.69 | 63.1 | 5.32 |
| 10% | 0.604 | 15.33 | 67.7 | 6.26 |
| 15% | 0.60 | 14.25 | 67.1 | 5.74 |

Table S8. Sn²⁺ and Sn⁴⁺ ratios with varying PEAI contents.

|  | 5% PEAI | 10% PEAI | 15% PEAI |
| --- | --- | --- | --- |
| Sn2+ | 83.04% | 84.27% | 83.43% |
| Sn4+ | 16.96% | 15.73% | 16.57% |

Table S9. Efficiency data with different TMSO concentrations.

| TMSO conc. (μL) | Voc (V) | Jsc (mA cm-2) | FF | PCE (%) |
| --- | --- | --- | --- | --- |
| 0 | 0.605 | 15.518 | 69.0 | 6.48 |
| 5 | 0.590 | 16.781 | 67.2 | 6.65 |
| 15 | 0.594 | 19.46 | 72.1 | 8.33 |
| 25 | 0.595 | 18.312 | 70.0 | 7.63 |

Table S10. Sn²⁺ and Sn⁴⁺ ratios with varying TMSO contents.

|  | 0μL TMSO | 5μL TMSO | 15μL TMSO | 25μL TMSO |
| --- | --- | --- | --- | --- |
| Sn2+ | 84.27% | 86.84% | 93.78% | 92.08% |
| Sn4+ | 15.73% | 13.16% | 6.22% | 7.92% |

Table S11. Photoluminescence lifetime parameters of perovskites under different large organic cation conditions.

|  | PEAI | BDEA | BAI | BATo |
| --- | --- | --- | --- | --- |
| $\boldsymbol{\tau}_{\mathbf{1}}$ | 1.28 | 1.66 | 1.52 | 1.98 |
| $\boldsymbol{\tau}_{\mathbf{2}}$ | 3.08 | 3.81 | 4.32 | 5.25 |
| A1(%) | 0.825 | 0.813 | 0.769 | 0.785 |
| A2(%) | 0.175 | 0.187 | 0.231 | 0.215 |
| Lifetime(ns) | 1.88 | 2.40 | 2.81 | 3.35 |

Table S12. Sn²⁺ and Sn⁴⁺ ratios under different large organic cation conditions.

|  | PEAI | BDEA | BAI | BATo |
| --- | --- | --- | --- | --- |
| Sn2+ | 93.76% | 94.78% | 96.34% | 96.78% |
| Sn4+ | 6.24% | 5.22% | 3.66% | 3.22% |

Table S13. Device efficiency data with PEAI, BAI, BATo, and BDEA addition.

| Organic cation | Voc (V) | Jsc (mA cm-2) | FF | PCE (%) |
| --- | --- | --- | --- | --- |
| BAI | 0.614 | 19.86 | 72.4 | 8.84 |
| BATo | 0.702 | 20.10 | 72.2 | 10.18 |
| BDEA | 0.627 | 18.79 | 72.4 | 8.53 |
| PEAI | 0.605 | 19.33 | 71.6 | 8.37 |

Table S14. DFT-calculated binding energies of intermolecular interactions among FA, BATo, and SnI₂.

| **Intermolecular interactions** | **Binding energy E_b_ (eV)** |
| --- | --- |
| FA-SnI2 | −1.197 |
| FA-BATO | −1.526 |
| SnI2-BATO | −1.434 |
| BATO-FA-SnI2 | -1.710 |

Table S15. DFT-calculated binding energies of Sn²⁺–solvent complexes for TMSO, DMSO, and DMF.

| **Ligand** | **Binding energy E_b_ (eV)** |
| --- | --- |
| TMSO | −1.103 |
| DMSO | −1.075 |
| DMF | −1.054 |

Table S16. Electronic energy levels and optical bandgaps of perovskite films modified with different bulky cations.

| **Sample** | **E_F_ (eV)** | **VBM (eV)** | **Optical E_g_ (eV)** | **CBM (eV)** |
| --- | --- | --- | --- | --- |
| PEAI | -4.83 | -5.53 | 1.39 | -4.14 |
| BAI | -4.74 | -5.53 | 1.40 | -4.13 |
| BDEA | -4.76 | -5.61 | 1.42 | -4.19 |
| BATo | -4.62 | -5.55 | 1.40 | -4.15 |

Table S17. EIS fitting parameters of perovskite solar cells incorporating different bulky-cations.

| **Sample** | **R_s_ (Ω cm^2^)** | **R_rec_ (kΩ cm^2^)** | **CPE-T (µF cm^−2^)** | **CPE-n** | **τ_rec_ (μs)** |
| --- | --- | --- | --- | --- | --- |
| PEAI | 13.14 | 1330 | 1.9491E-08 | 0.98 | 0.0259 |
| BDEA | 15.61 | 1394 | 2.04E-08 | 0.99 | 0.0284 |
| BAI | 10.93 | 1497 | 2.04E-08 | 0.99 | 0.0305 |
| BATo | 12.9 | 1632 | 1.9582E-08 | 0.95127 | 0.0320 |

Table S18 . Quantitative KPFM analysis of surface potential distribution and grain-boundary potential contrast in pristine and passivated perovskite films.

| **Sample** | **Mean V_CPD_ (mV)** | **V_CPD_ on grain (mV)** | **V_CPD_ at GB (mV)** | **ΔV grain–GB (mV)** | **σ(V_CPD_) (mV)** |
| --- | --- | --- | --- | --- | --- |
| Pristine | -88 | -79 | -95 | 16 | 8.54 |
| PEAI | -19.1 | -15 | -30 | 15 | 5.00 |
| BATo | 4.39 | 10 | -1 | 11 | 3.90 |

**Reference**

[1]	P. E. Blöchl, *Physical review B* **1994**, 50, 17953.

[2]	a)J. P. Perdew, W. Yue, *Physical review B* **1986**, 33, 8800; b)J. P. Perdew, J. A. Chevary, S. H. Vosko, K. A. Jackson, M. R. Pederson, D. J. Singh, C. Fiolhais, *Physical review B* **1992**, 46, 6671.

[3]	C. Laurence, J.-F. Gal, *Lewis basicity and affinity scales: data and measurement*, John Wiley & Sons, **2009**.

[4]	S. Wang, Y. Chen, R. Li, Y. Xu, J. Feng, D. Yang, N. Yuan, W. H. Zhang, S. Liu, J. Ding, *Advanced Science* **2020**, 7, 1903009.

[5]	Q. Zhou, R. Guo, S. Liu, N. Li, M. Xu, X. Zhang, X. Jiang, L. Wang, L.-I. Dion-Bertrand, Z. Shi, *Nature Energy* **2026**, 1.
